# The Transcription Factor Pdr802 Regulates Titan Cell Formation and Pathogenicity of Cryptococcus neoformans

**DOI:** 10.1128/mBio.03457-20

**Published:** 2021-03-09

**Authors:** Julia C. V. Reuwsaat, Daniel P. Agustinho, Heryk Motta, Andrew L. Chang, Holly Brown, Michael R. Brent, Livia Kmetzsch, Tamara L. Doering

**Affiliations:** a Molecular Biology of Pathogens Laboratory, Biotechnology Center, Universidade Federal do Rio Grande do Sul, Porto Alegre, Brazil; b Department of Molecular Microbiology, Washington University School of Medicine, St. Louis, Missouri, USA; c Center for Genome Sciences and Systems Biology, Washington University School of Medicine, St. Louis, Missouri, USA; d Department of Computer Science and Engineering, Washington University, St. Louis, Missouri, USA; e Department of Genetics, Washington University, St. Louis, Missouri, USA; f Department of Molecular Biology and Biotechnology, Universidade Federal do Rio Grande do Sul, Porto Alegre, Brazil

**Keywords:** *Cryptococcus neoformans*, Pdr802, Titan cells, capsule, fungal pathogens, pathogenesis, pathogenic yeast, quorum sensing, transcription factors

## Abstract

Cryptococcus neoformans is a ubiquitous, opportunistic fungal pathogen that kills almost 200,000 people worldwide each year. It is acquired when mammalian hosts inhale the infectious propagules; these are deposited in the lung and, in the context of immunocompromise, may disseminate to the brain and cause lethal meningoencephalitis. Once inside the host, C. neoformans undergoes a variety of adaptive processes, including secretion of virulence factors, expansion of a polysaccharide capsule that impedes phagocytosis, and the production of giant (Titan) cells. The transcription factor Pdr802 is one regulator of these responses to the host environment. Expression of the corresponding gene is highly induced under host-like conditions *in vitro* and is critical for C. neoformans dissemination and virulence in a mouse model of infection. Direct targets of Pdr802 include the quorum sensing proteins Pqp1, Opt1, and Liv3; the transcription factors Stb4, Zfc3, and Bzp4, which regulate cryptococcal brain infectivity and capsule thickness; the calcineurin targets Had1 and Crz1, important for cell wall remodeling and C. neoformans virulence; and additional genes related to resistance to host temperature and oxidative stress, and to urease activity. Notably, cryptococci engineered to lack Pdr802 showed a dramatic increase in Titan cells, which are not phagocytosed and have diminished ability to directly cross biological barriers. This explains the limited dissemination of *pdr802* mutant cells to the central nervous system and the consequently reduced virulence of this strain. The role of Pdr802 as a negative regulator of Titan cell formation is thus critical for cryptococcal pathogenicity.

## Abstract (precis)

The pathogenic yeast Cryptococcus neoformans presents a worldwide threat to human health, especially in the context of immunocompromise, and current antifungal therapy is hindered by cost, limited availability, and inadequate efficacy. After the infectious particle is inhaled, C. neoformans initiates a complex transcriptional program that integrates cellular responses and enables adaptation to the host lung environment.

## INTRODUCTION

Cryptococcosis is a fungal infection caused by Cryptococcus neoformans and Cryptococcus gattii. C. neoformans is a ubiquitous opportunistic pathogen that infects mainly immunocompromised patients, while C. gattii is capable of infecting immunocompetent individuals (1). Cryptococcosis causes 180,000 deaths worldwide each year, including roughly 15% of all AIDS-related deaths (2), and is initiated by the inhalation of spores or desiccated yeast cells. In immunocompetent individuals, this typically causes an asymptomatic pulmonary infection that is controlled by the host immune response, although a population of C. neoformans may remain latent for extended periods of time (3–5).

Under conditions of immunocompromise, cryptococci disseminate from the lung to the brain. Mechanisms that have been suggested to mediate fungal crossing of the blood-brain barrier (BBB) include transcellular migration, in which the yeast cells enter and exit vascular endothelial cells (6–9); paracellular movement, in which they cross the BBB at junctions between endothelial cells (10–12); and “Trojan horse” crossing, whereby macrophages harboring C. neoformans enter the brain (13). Cryptococcal meningoencephalitis is difficult to treat and frequently lethal, for reasons that include the availability and cost of therapy (14, 15).

The ability of C. neoformans to survive and proliferate in the lung, and subsequently disseminate to the brain, depends on viability at mammalian body temperature and the expression of multiple virulence traits; these include secreted factors (16, 17), a polysaccharide capsule that surrounds the cell wall (18), and the production of giant (Titan) cells (19, 20). One secreted molecule, the pigment melanin, associates with the cell wall, where its antioxidant properties protect fungal cells from reactive oxygen species produced as a host immune defense (21–25). Urease, a secreted metalloenzyme that converts urea to ammonia and CO_2_, may affect the course of infection by modulating environmental pH and damaging host tissue structure (11, 12, 26).

The capsule, composed primarily of large polysaccharides (27–29), is a key cryptococcal virulence factor that impairs phagocytosis by immune cells (30–35). This dynamic entity changes its size and structure during interactions with the host or external environment (36–39), contributing to fungal adaptation (40, 41). Capsule polysaccharides that are shed from the cell enable diagnosis of cryptococcal infection and also impede host responses (35, 42).

Titan cells are a cryptococcal morphotype that has been variously characterized as having a cell body diameter (excluding the capsule) greater than 10 or 15 μm or total cell diameter (including the capsule) that exceeds 30 μm (20, 43, 44). These cells are polyploid and produce normal-size cells during infection (19, 45, 46). Titan cell formation is triggered by exposure to the host environment, including nutrient starvation, reduced pH, and hypoxia (47–49), although the extent of induction depends on the host immune response and the duration of infection (45, 50). Titan cell production appears to benefit the development of pulmonary C. neoformans infection, since these large cells are less susceptible to internalization by host phagocytes and more resistant to oxidative stress than normal-size cells (19, 46). Some of these effects may be explained by the highly cross-linked capsule and thickened cell wall of Titan cells (51). In contrast to their success in the lungs, Titan cells show impaired dissemination to the brain (19, 46).

C. neoformans experiences a dramatic change in conditions upon entering a host, including altered nutrient levels and pH. To adapt to the new environment, cryptococci activate a network of transcription factors (TFs) (39, 52). For example, imbalances in ion homeostasis trigger transcriptional changes mediated by the TFs Zap1 (53), Cuf1 (54), Pho4 (55), Cir1 (56), and Crz1 (57). Alkaline pH stimulates expression of the TF Rim101, which enables growth under basic conditions and other stresses, such as high salt and iron limitation; it also promotes the association of capsule polysaccharide with the cell and the formation of Titan cells (47, 58).

Overlapping TF circuits regulate cryptococcal virulence determinants, including polysaccharide capsule production and melanin synthesis. For example, Usv101, an important regulator of capsule thickness and polysaccharide shedding, also regulates three other TFs (Gat201, Crz1, and Rim101) and multiple polysaccharide-related enzymes (59). Gat201 further regulates additional virulence-related transcription factors and the anti-phagocytic protein Blp1 (60), while Crz1 plays a central role in the maintenance of plasma membrane and cell wall stability (57, 61, 62). Crz1 expression is also modulated by the calcineurin signaling pathway, which is required for normal yeast growth at 37°C, virulence, and sexual reproduction (63). A group of TFs, including Usv101, Bzp4, Hob1, and Mbs1 (59, 64), act together to regulate melanin production; deletion of Bzp4 also alters capsule production (52).

In this study, we investigated the TF Pdr802. The corresponding gene has a high rate of nonsynonymous mutations, which suggests that it is evolving rapidly (65). Pdr802 has previously been implicated in C. neoformans virulence (39, 52, 66), but its specific role and targets are not known. We discovered that Pdr802 is induced under host-like conditions, is a negative regulator of Titan cell formation, and influences capsule thickness and phagocytosis by macrophages. It also regulates genes whose products act in cell wall remodeling, virulence factor production, resistance to host temperature and oxidative stress, and quorum sensing. These functions make Pdr802 critical for cryptococcal survival in the lung and dissemination to the brain.

## RESULTS

### Role of Pdr802 in C. neoformans virulence.

The importance of Pdr802 in C. neoformans virulence has been demonstrated in multiple experimental models. Liu and collaborators first reported in 2008 that partial deletion of *PDR802* reduced C. neoformans infectivity in a competition assay of pooled C. neoformans strains (66). In 2015, Maier et al. showed that a *pdr802* deletion mutant had reduced virulence when tested individually in a short-term mouse model of infection (39). Later that year, Jung and colleagues reported that Pdr802 was required for full virulence in both wax moth larva and short-term mouse infection using pooled strains (52). Most recently, Lee and collaborators showed that Pdr802 was required for brain infection (67).

To further investigate the role of Pdr802 in pathogenesis, we complemented a complete deletion strain in the KN99α background that we had previously generated (*pdr802*) (39) with the intact gene at its native locus (*PDR802*). To examine targets of Pdr802, we also constructed a strain that expresses the protein fused to mCherry at its N terminus (see Fig. S1A in the supplemental material). All of these strains lacked or expressed RNA encoding *PDR802* or its modified forms as expected (Fig. S1B), and *PDR802* was expressed at wild-type levels in the complemented and modified strains (Fig. S1C).

10.1128/mBio.03457-20.1 FIG S1 Mutant strain construction and confirmation. (A) Scheme for generating C. neoformans strains in the KN99α background (middle) that either lack *PDR802* (*pdr802*, top) or encode a tagged copy of the protein (*mCherry-PDR802*). (B) Qualitative analysis of gene expression in panel A strains and the complemented *pdr802* mutant (*PDR802*). Cryptococcal mRNA isolated from cells grown in DMEM (37°C, 5% CO_2_, 24 h) was used to generate cDNA; from this, segments of the genes indicated at the left were amplified using the primers listed in Data Set S2, sheet 5, and the products were analyzed by agarose gel electrophoresis. Fragment sizes (in base pairs) are indicated, and the ladder bands shown are 400, 500, 650, 850, and 1,000 bp for the top panel; 200, 300, 400, 500, and 650 bp for the middle panel; and 100, 200, and 300 bp for the bottom panel. (C) Quantitative analysis of *PDR802* expression. Samples of RNA isolated as for panel B were analyzed for *PDR802* expression by qRT-PCR. All results were normalized to *ACT1* expression. Each symbol represents a biological replicate, with the mean and standard deviation also shown. ***, *P* < 0.001, compared to KN99α by one-way ANOVA with a *post hoc* Dunnett test. Download FIG S1, PDF file, 0.5 MB. Copyright © 2021 Reuwsaat et al. 2021 Reuwsaat et al. https://creativecommons.org/licenses/by/4.0/ This content is distributed under the terms of the Creative Commons Attribution 4.0 International license.

**FIG 1:**
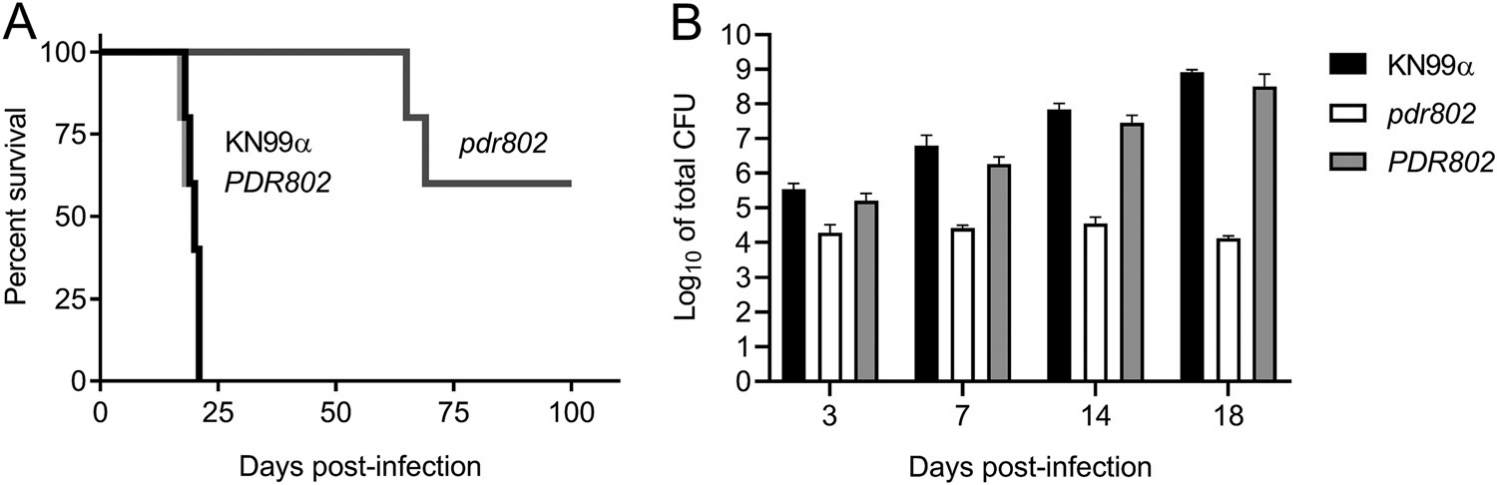
The transcription factor Pdr802 influences C. neoformans virulence. (A) Survival of C57BL/6 mice over time after intranasal inoculation with 5 × 10^4^ cryptococci, with sacrifice triggered by weight below 80% of peak. (B) Means and standard deviations (SD) of total colony-forming units (CFU) in lung tissue at various times post-infection. Numbers of CFU inoculated for each strain were 41,400 (KN99α), 42,800 (*pdr802* strain) and 26,600 (*PDR802* strain). *P* < 0.05 for the *pdr802* strain compared to the other strains at all time points.

We next assessed the long-term survival of C57BL/6 mice infected with the parental wild-type (WT) strain (KN99α), the deletion mutant (*pdr802*), or the complemented mutant (*PDR802*). In this model, mice infected with the parent or complemented strains survived for roughly 3 weeks, while those infected with the deletion mutant showed a striking increase in survival: all animals survived for at least 65 days, and over half survived to the end of the study (100 days) (Fig. 1A). The lung burden measured at the time of death for *pdr802*-infected mice in this study was approximately 100-fold lower than that of wild-type infections (Fig. S2A), demonstrating the importance of this TF in C. neoformans virulence. Mean brain burden at the time of death was more similar between mutant and wild-type infections (Fig. S2A), although we did note some heterogeneity in this measure for *pdr802*-infected mice; animals sacrificed at around 2 months of infection (red symbols) showed brain burdens similar to WT levels, while brain burdens in mice sacrificed at day 100 (blue symbols) ranged between zero fungal cells and the WT level.

10.1128/mBio.03457-20.2 FIG S2 Organ burdens. (A) Mean and SD values of total CFU in the indicated tissue of mice from the Fig. 1 survival curve are shown. Each point is the average value for a single animal at the time of death. For *pdr802* strain infections, red circles represent mice sacrificed at days 65 and 69, while blue circles represent mice sacrificed at the termination of the study (day 100). (B) Mean and SD of total CFU in the blood and brain at the indicated times post-infection. (C) Mean and SD of total CFU in the lung, blood, and brain 75 days after infection with the *pdr802* strain. Each color represents one mouse. Download FIG S2, PDF file, 0.9 MB. Copyright © 2021 Reuwsaat et al. 2021 Reuwsaat et al. https://creativecommons.org/licenses/by/4.0/ This content is distributed under the terms of the Creative Commons Attribution 4.0 International license.

We next examined the time course of fungal proliferation in the lungs. As expected, the burdens of WT and the complemented mutant strains increased steadily over an 18-day interval (Fig. 1B), eventually reaching roughly 10^5^ times the original inoculum. Towards the end of this period, these cells were also detected in the blood and brain (Fig. S2B). In contrast, the lung burden of *pdr802* remained close to the inoculum throughout this period, with no mutant cells detected in the blood or brain. At a late time point of *pdr802* infection (75 days), we again noted some heterogeneity of fungal burden: one mouse had high lung burden with no dissemination, another had high lung burden with moderate brain burden, and the third had extremely low lung burden with no dissemination (Fig. S2C). No CFU were detected in the blood of *pdr802*-infected mice at any point during infection. These results suggest that even though the *pdr802* mutant is generally hypovirulent and remains at low levels in the lung, it can occasionally reach the brain and, given enough time, accumulate there (see Discussion).

Given the dramatic effects of Pdr802 on fungal virulence, we wondered about the specific biological processes in which this transcription factor is involved. We first examined the behavior of the *pdr802* strain *in vitro*, including stress conditions that might be encountered in the host. We saw no differences in growth of the mutant compared to WT cells under conditions that challenge cell or cell wall integrity, including the presence of sorbitol, high salt, cell wall dyes, caffeine, sodium dodecyl sulfate (SDS), or ethanol (Fig. S3A to C). The mutant also showed no altered susceptibility to elements of the host response, such as nitrosative or oxidative stresses, or in melanin production. All of these results held when cells were grown on rich medium, whether plates were incubated at 30°C, 37°C, or 37°C in the presence of 5% CO_2_, which was recently described as an independent stress for C. neoformans (68) (Fig. S3A to C). Finally, the mutant showed no difference from wild-type cells in secretion of urease (Fig. S3D).

10.1128/mBio.03457-20.3 FIG S3 Characterization of *pdr802* cells. (A to C) Tenfold serial dilutions of WT, *pdr802*, and *PDR802* cells were plated on the media shown and incubated at 30°C (A), 37°C (B), or 37°C in the presence of 5% CO_2_ (C). Nitrosative (NaNO_2_) and oxidative (H_2_O_2_) stress plates were prepared with YNB medium, and melanization plates containing l-DOPA were prepared as described in Materials and Methods; all other plates were prepared with YPD medium. The *lac1* control strain lacks the ability to melanize (88). (D) Urease activity of the indicated strains was evaluated using Christensen’s urea solid medium (see Materials and Methods) at the indicated temperatures. The *ure1* control strain does not produce urease (17). Download FIG S3, PDF file, 0.8 MB. Copyright © 2021 Reuwsaat et al. 2021 Reuwsaat et al. https://creativecommons.org/licenses/by/4.0/ This content is distributed under the terms of the Creative Commons Attribution 4.0 International license.

### Pdr802 is regulated by “host-like” conditions.

**FIG 2:**
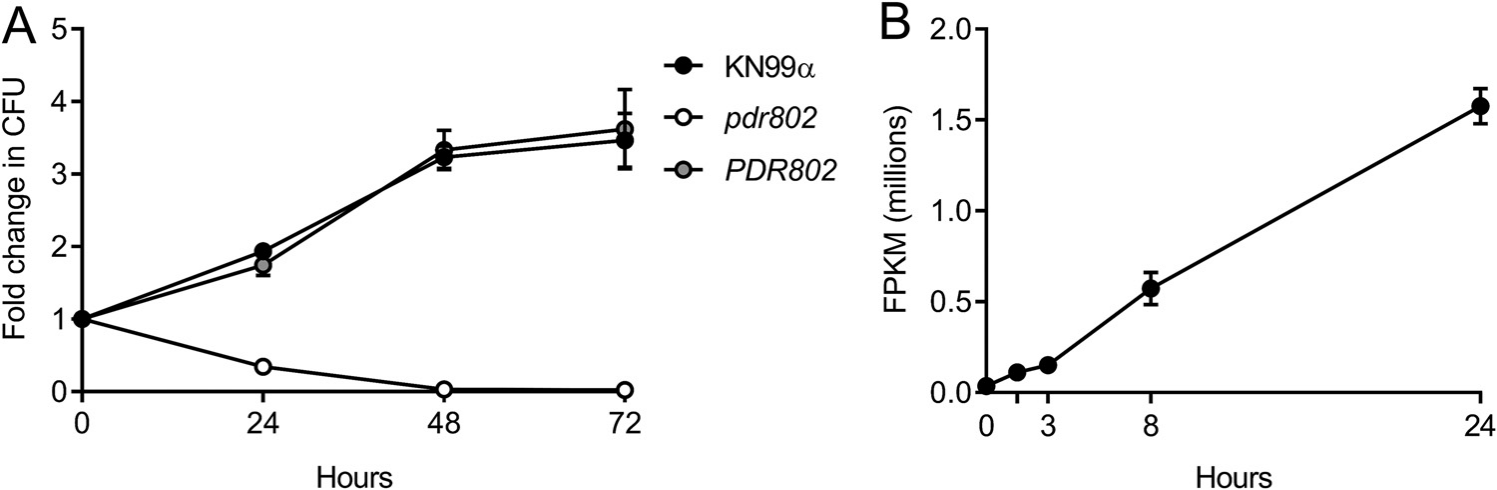
*PDR802* expression is required for cell viability and induced during growth under host-like conditions. (A) Cells grown in DMEM at 37°C and 5% CO_2_ were sampled at the times indicated and plated on YPD to assess viability (measured as number of CFU and plotted as fold change from time zero). (B) *PDR802* expression in KN99α cells grown in DMEM at 37°C and 5% CO_2_ was assessed by RNA-Seq as in reference 117. FPKM, fragments per kilobase per million. Mean and standard deviation (SD) are shown on both plots.

We next tested the growth of the *pdr802* mutant under conditions more like those encountered inside the mammalian host, using tissue culture medium (Dulbecco’s modified Eagle medium [DMEM]) at 37°C in the presence of 5% CO_2_. We found that although the *pdr802* mutant grew like WT in rich medium (yeast extract-peptone-dextrose [YPD]), it grew poorly in DMEM (Fig. S4A and B). To test whether the mutant cells were dead or just static after growth in DMEM, we plated aliquots on solid medium to measure CFU over time (Fig. 2A). The *pdr802* culture showed a dramatic decrease in viability compared to WT and the complemented strain, which was greatest in the first 24 h. This is the same time frame in which expression of the *PDR802* gene shows a striking increase, as measured by transcriptome sequencing (RNA-Seq) (Fig. 2B).

10.1128/mBio.03457-20.4 FIG S4 Growth curves and capsule shedding. (A and B) Growth of the indicated strains in YPD at 30°C (A) or DMEM at 37°C and 5% CO_2_ (B) was assessed by determining the optical density at 600 nm (OD_600_) at the times indicated. (C) Conditioned medium from the indicated strains was probed for the presence of GXM after growth in DMEM for 24 or 48 h. Equal volumes of culture supernatant were analyzed without normalization to cell density. Immunoblotting was performed using the anti-GXM monoclonal antibody 302. Download FIG S4, PDF file, 0.6 MB. Copyright © 2021 Reuwsaat et al. 2021 Reuwsaat et al. https://creativecommons.org/licenses/by/4.0/ This content is distributed under the terms of the Creative Commons Attribution 4.0 International license.

**FIG 3:**
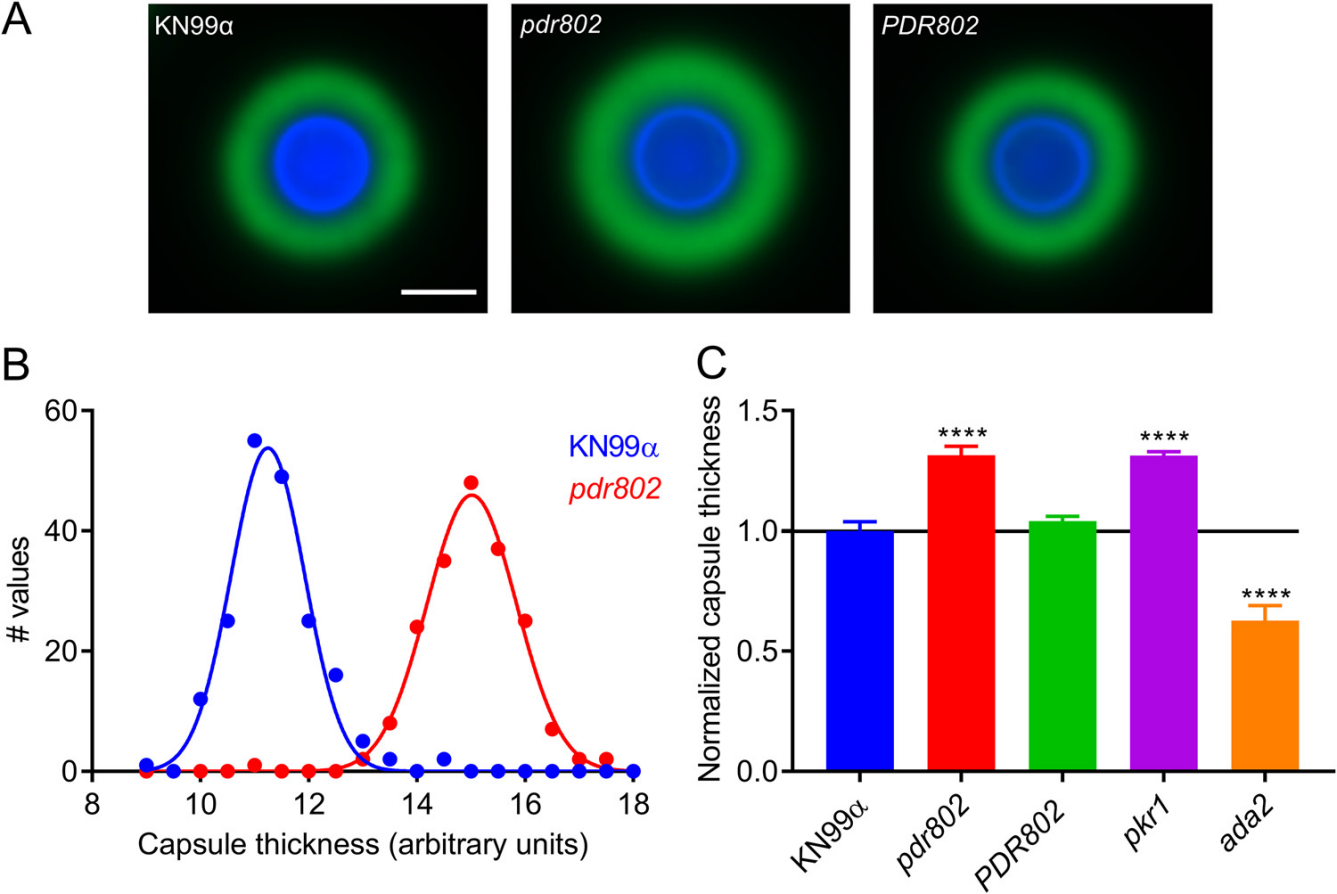
The *pdr802* mutant is hypercapsular. (A) Representative immunofluorescence micrographs of the indicated strains after growth in DMEM (37°C, 5% CO_2_) for 24 h. The capsule was stained with anti-GXM monoclonal antibody 302 conjugated with Alexa Fluor 488 (green) and the cell wall with calcofluor white (blue). All images are to the same scale; bar, 5 μm. (B) Capsule thickness distribution for the indicated strains. (C) Mean and SD of capsule size, quantified as detailed in Materials and Methods and Fig. S5, with the *pkr1* (39) and *ada2* (105) strains shown as hypercapsular and hypocapsular controls, respectively. ****, *P* < 0.0001, compared to KN99α by one-way ANOVA with a *post hoc* Dunnett test.

Another important feature that is induced by growth in DMEM at 37°C and 5% CO_2_ is the polysaccharide capsule, which we previously reported to be regulated by Pdr802, based on negative staining with India ink (39). Fluorescence microscopy confirmed increased capsule thickness of the mutant, which reverted to WT in the complemented strain (Fig. 3A). To quantify this change, we took advantage of a semiautomated assay that we have developed (Fig. S5), which measures capsules on a population scale (Fig. 3B) and is therefore very sensitive. This analysis showed that the capsule thickness of *pdr802* cells resembles that of the well-studied hypercapsular mutant *pkr1* (39, 69, 70) and is completely restored to WT by complementation at the native locus (Fig. 3C). Previous studies suggest that capsule thickness upon induction reflects the size of the dominant capsule polymer (glucuronoxylomannan [GXM]) (71, 72), which can be analyzed by agarose gel migration and blotting with anticapsule antibodies (71). Consistent with the difference we observed in capsule thickness by imaging, this method showed decreased mobility of GXM from *pdr802* as capsule induction progressed (Fig. S4C).

10.1128/mBio.03457-20.5 FIG S5 Semi-automated assay for cryptococcal capsule imaging. (A) Schematic of applying this method to cryptococcal cells induced to form capsule by growth in DMEM (37°C, 5% CO_2_) for 24 h, followed by cell wall and capsule staining. Thousands of cells may be imaged per well and analyzed automatically with software that annotates and measures the capsule (annotated in dark blue) and cell wall (annotated in bright green). See Materials and Methods for details. (B) Capsule size distribution of WT cells after induction. Capsule thickness for each cell is the difference between the paired diameters of the cell wall and capsule, which is plotted with reference to the mean value. (C and D) Mean and SD (C) and cumulative percentage (D) analysis of WT compared to hyper- and hypocapsular control strains (here, *pkr1* and *ada2*, respectively). Capsule thickness is in arbitrary units, related to the pixels measured. (E) Time required to analyze the capsule thickness of 1,000 cells by this method compared to manual assessment of India ink images. Download FIG S5, PDF file, 0.7 MB. Copyright © 2021 Reuwsaat et al. 2021 Reuwsaat et al. https://creativecommons.org/licenses/by/4.0/ This content is distributed under the terms of the Creative Commons Attribution 4.0 International license.

**FIG 4:**
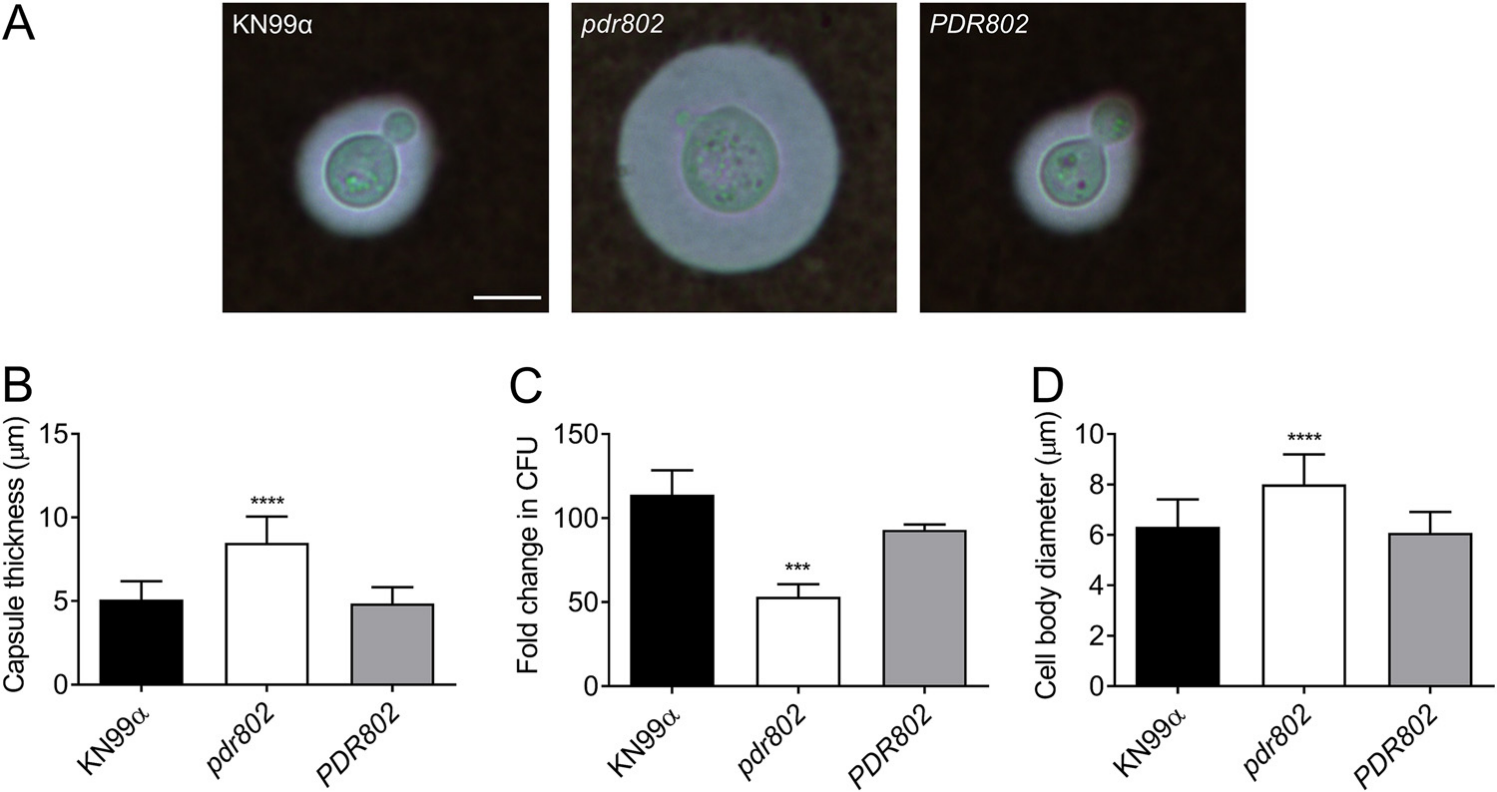
Growth in mouse serum elicits increased capsule thickness and cell body diameter in the *pdr802* mutant. (A) Light micrographs of the indicated strains after growth in mouse serum (at 37°C, 5% CO_2_) for 24 h and negative staining with India ink to visualize the capsule. All images are to the same scale; bar, 5 μm. (B) Mean and SD of capsule thickness, assessed by measuring at least 50 cells per strain with ImageJ. (C) Cells grown as described for panel A were plated on YPD to assess CFU. Mean and SD of the fold change compared to 0 h are shown. (D) Mean and SD of cell body diameter, measured as for panel B. ***, *P* < 0.001, and ****, *P* < 0.0001, compared to KN99α by one-way ANOVA with a *post hoc* Dunnett test.

To validate the observations that we had made under standard host-like conditions based on synthetic tissue culture medium, we conducted similar studies in mouse serum at 37°C and 5% CO_2_. These conditions induced an even more pronounced hypercapsular phenotype of the *pdr802* mutant (Fig. 4A and B), as well as reduced cell viability (Fig. 4C) and increased cell body diameter (Fig. 4D).

**FIG 5:**
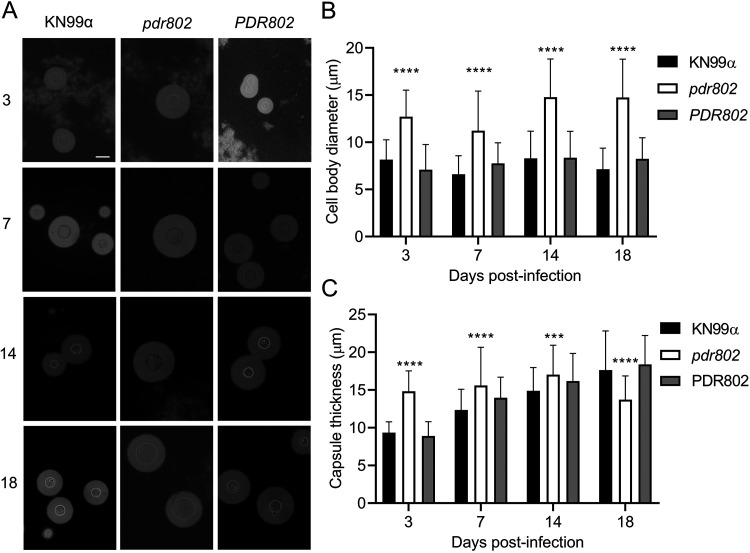
Absence of *PDR802* yields enlarged cells and loss of capsule induction in the context of animal infection. (A) India ink staining of fungi isolated from the lungs of mice infected with the indicated strains. Numbers at left are days post-infection. All images are to the same scale; bar, 10 μm. (B and C) Means and SD of cell body diameter (B) and capsule thickness (C), assessed by measuring at least 50 cells per strain with ImageJ. ****, *P* < 0.0001, and ***, *P* < 0.001, compared to KN99α by one-way ANOVA with a *post hoc* Dunnett test for each day post-infection.

We were intrigued by the enlarged cell body and capsule of the *pdr802* mutant cells under host-like conditions *in vitro* and decided to examine these phenotypes *in vivo*. For these studies, we isolated fungal cells from the lungs of mice at various times after infection and assessed their morphology by negative staining (Fig. 5A). At each time point, the mean mutant cell body diameter was larger than that of the controls. Additionally, while this parameter was stable for WT and complemented strains throughout the infection period, it trended larger at the end of the infection period for the deletion mutant (Fig. 5B). In contrast, mutant capsule thickness, although initially greater than that of control cells, changed little throughout the period, while capsule thickness of control cells increased to that level or beyond (Fig. 5C). Furthermore, although the total diameter of *pdr802* cells consistently exceeded that of WT and complemented cells, their sizes became more comparable late in infection (Fig. S6A). Over time, therefore, the ratio of total cell diameter to cell body diameter for WT and *PDR802* cells steadily increased, while it remained roughly constant for the mutant (Fig. S6B).

10.1128/mBio.03457-20.6 FIG S6 *PDR802* deletion induces the formation of Titan cells. Mean and SD of (A) total cell diameter and (B) ratio of total cell to cell body diameters (diameter ratio), assessed by measuring at least 50 cells per strain with ImageJ. **, *P* < 0.01, and ****, *P* < 0.0001, compared to KN99α by one-way ANOVA with a *post hoc* Dunnett test for each day post-infection. (C) Percent of Titan cells in the indicated strain, evaluated using various published parameters: cell body diameter bigger than 10 or 15 μm (20) or total cell diameter bigger than 30 μm (43). Download FIG S6, PDF file, 0.7 MB. Copyright © 2021 Reuwsaat et al. 2021 Reuwsaat et al. https://creativecommons.org/licenses/by/4.0/ This content is distributed under the terms of the Creative Commons Attribution 4.0 International license.

### Pdr802 negatively regulates Titan cell formation.

We were particularly interested in the cell size phenotype of *pdr802* because Titan cells have been strongly implicated in cryptococcal pathogenesis (19). By any definition of this morphotype (cell body diameter greater than 10 or 15 μm or total cell diameter greater than 30 μm), our mutant cell populations were dramatically enriched in Titan cells at every time of infection that we assessed (Fig. S6C).

**FIG 6:**
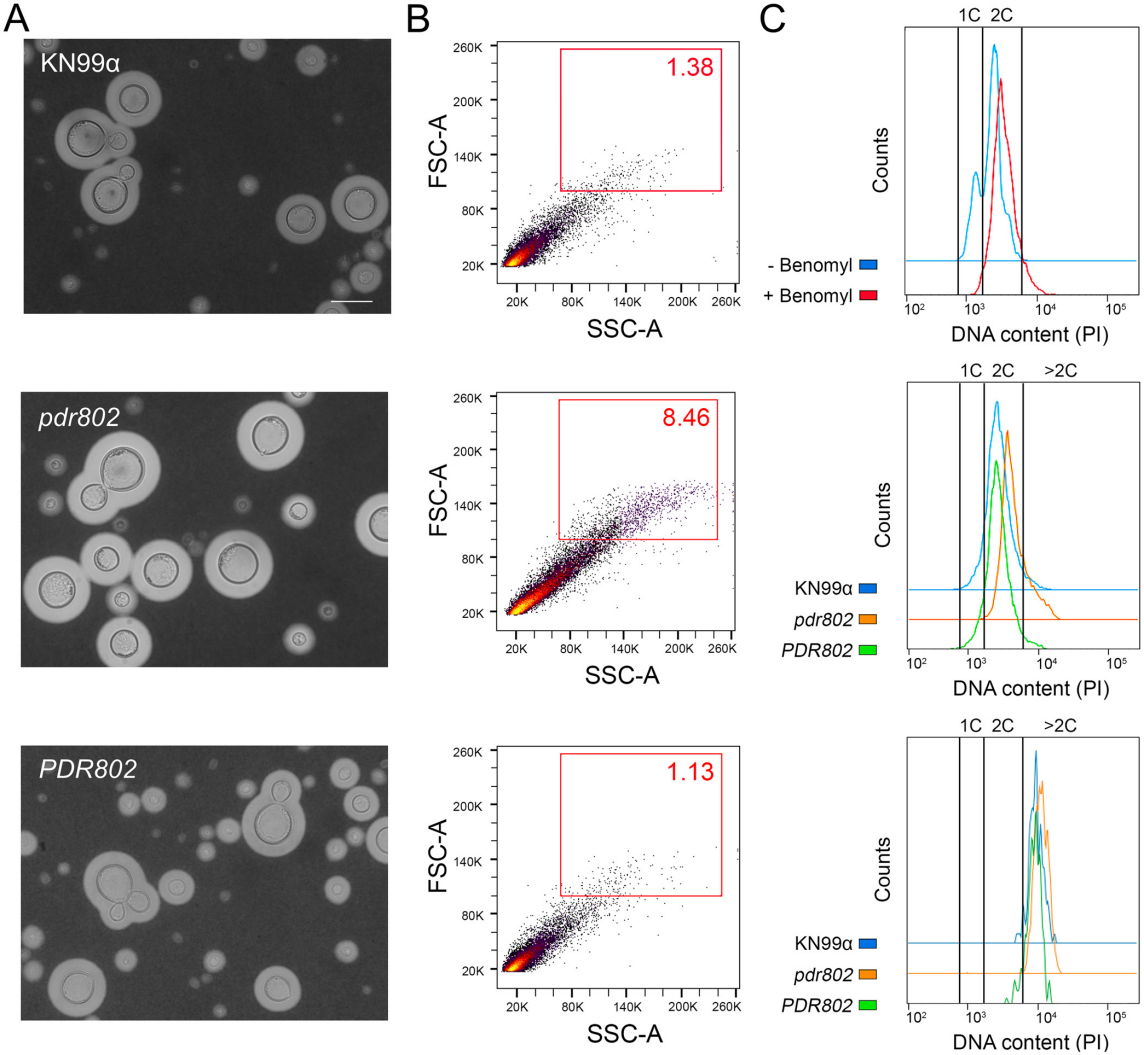
Pdr802 is a negative regulator of Titan cell formation. (A) The indicated strains were subjected to *in vitro* conditions that induce Titan cell formation and imaged with India Ink. All images are to the same scale; bar, 10 μm. Images were selected so that each shows multiple examples of Titan cells, not to reflect abundance of this morphotype. (B) The percentage of Titan cells in each culture was quantified using flow cytometry, gated as indicated by the red square. FSC-A, forward scatter; SSC-A, side scatter. (C) DNA content of cells after staining with propidium iodide (PI) and analysis by flow cytometry. (Top) 1C and 2C gates determined using control cultures of KN99α grown in YNB without or with benomyl, which traps cells at 2C. (Middle) Profiles of the indicated strains after growth under Titan cell-inducing conditions, showing an increased polyploid tail in mutant cultures relative to wild-type cells. (Bottom) DNA content profiles for only the Titan cells (gated as for panel B, red box) for each strain, showing their polyploid nature.

To specifically test Titan cell formation by the *pdr802* strain, we subjected mutant cells to *in vitro* conditions that induce this process (49) (Fig. 6A) and analyzed the resulting population by flow cytometry. Consistent with our *in vivo* observations, Titan cells constituted a much larger fraction of the population in the mutant culture (8.46%) than in the WT and complemented cultures (1.38% and 1.13%, respectively) (Fig. 6B). As expected for Titan cells, these cells are also polyploid (Fig. 6C).

**FIG 7:**
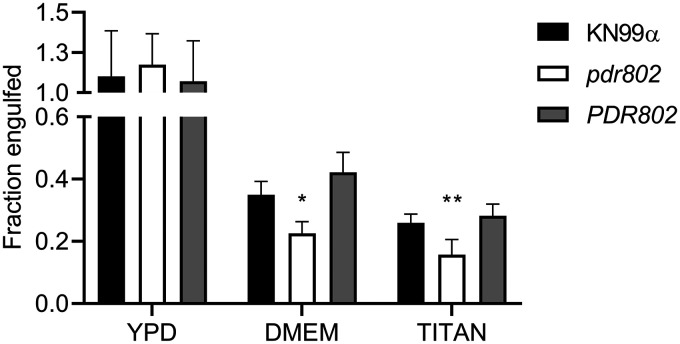
Deletion of *PDR802* affects phagocytosis after growth under conditions that induce capsule and Titan cell formation. C. neoformans strains were grown in YPD (18 h), DMEM (24 h), or Titan cell induction medium (72 h) and then incubated for 2 h with J774.16 mouse macrophages; host cells were then washed and lysed to assess fungal burden by CFU. Data shown are normalized to the CFU of the initial inoculum. *, *P* < 0.05, and **, *P* < 0.01, compared to KN99α by one-way ANOVA with a *post hoc* Dunnett test.

Titan cells are poorly engulfed by host phagocytes (19, 45, 73), which may reflect their increased size as well as alterations in capsule and cell wall (51). We observed this reduced uptake for all strains after growth in conditions that favor Titan cell formation (Fig. 7, Titan versus YPD). Also, all strains showed a reduction in phagocytosis after capsule induction in DMEM (Fig. 7, DMEM versus YPD), which is not surprising, because the capsule is antiphagocytic (31, 73). Notably, the reduction in uptake was greatest for the *pdr802* mutant under both of these conditions, even though it showed normal engulfment when all strains were grown under control conditions (YPD). This is likely because the mutant culture is both hypercapsular and enriched in Titan cells.

### Identification of direct, functional targets of Pdr802.

To identify direct targets of Pdr802, we next performed chromatin immunoprecipitation followed by sequencing (ChIP-Seq). We compared DNA sequences immunoprecipitated by anti-mCherry monoclonal antibody (MAb) from cells expressing mCherry-Pdr802, which grow similarly to the WT (Fig. S7A), and untagged cells. Both strains were grown for 24 h in DMEM at 37°C and 5% CO_2_, as these conditions induce *PDR802* expression dramatically compared to standard YPD growth conditions (Fig. 2).

10.1128/mBio.03457-20.7 FIG S7 Strain viability, Pdr802 interaction with its own promoter, and putative DNA-binding motifs. (A) The indicated strains were grown in DMEM at 37°C and 5% CO_2_ for the times shown, and samples were tested for their ability to form colonies on YPD medium. Plotted is the fold change in CFU relative to the initial culture. (B) Pdr802 self-regulation. The ratios (log_2_) of reads from immunoprecipitated (IP) DNA to reads from input DNA were calculated for 1,000 bp upstream of the first coding nucleotide (+1) of *PDR802*; shown is the difference in these values between tagged and untagged strains. Red triangles, complete Pdr802 DNA-binding motifs (Fig. S7C); blue triangles, partial motifs. (C) Putative Pdr802-binding motifs determined using DREME (76). Primary and secondary hits are shown for analysis of 1,000 bp upstream of the initiating ATG. Download FIG S7, PDF file, 1.0 MB. Copyright © 2021 Reuwsaat et al. 2021 Reuwsaat et al. https://creativecommons.org/licenses/by/4.0/ This content is distributed under the terms of the Creative Commons Attribution 4.0 International license.

Using 2-fold-enrichment over control as a cutoff value for peaks with adjusted *P* values of <0.05, we identified 656 binding sites for mCherry-Pdr802 in genomic DNA. Of these, 540 occurred within 1,000 bp upstream of transcription start sites (Data Set S1, sheets 1 and 2), which we used as an approximation of regulatory regions. Notably, ChIP-Seq data for the region upstream of the *PDR802* transcription start site suggested self-regulation, as has been reported for other cryptococcal TFs (74, 75) (Fig. S7B). We further applied discriminative regular expression motif elicitation (DREME) (76) to the set of 540 upstream regions to identify putative Pdr802 binding motifs; these were highly enriched in GA (TC) (Fig. S7C).

10.1128/mBio.03457-20.8 DATA SET S1 Pdr802 ChIP-Seq, RNA-Seq, and dual-threshold optimization (DTO) data. Sheet 1, annotated peaks that occur in gene promoter regions that showed ≥2-fold enrichment when Chip-Seq was performed on strains expressing tagged versus untagged Pdr802. Sheet 2, ChIP-Seq primary data of Pdr802-specific peaks. Sheet 3, RNA-Seq data. Sheet 4, DTO filtered data. Sheet 5, DTO primary data. Sheet 6, ChIP-Seq data of all peaks in mCherry-Pdr802 samples. Sheet 7, ChIP-Seq data of all peaks in untagged (WT) control samples. Download Data Set S1, XLSX file, 2.5 MB. Copyright © 2021 Reuwsaat et al. 2021 Reuwsaat et al. https://creativecommons.org/licenses/by/4.0/ This content is distributed under the terms of the Creative Commons Attribution 4.0 International license.

To complement our ChIP studies, we determined the set of genes regulated by Pdr802 under host-like conditions by performing RNA-Seq of WT and *pdr802* cells after growth for 24 h in DMEM at 37°C and 5% CO_2_ (Data Set S1, sheet 3). We then used dual-threshold optimization (DTO) to analyze the RNA-Seq and ChIP-Seq data sets together. This statistical method allowed us to combine the evidence from binding and expression studies to converge on a set of direct and functional TF targets (77); because it uses two independent lines of evidence to suggest regulatory relationships, it increases confidence in highly significant small effects. The Pdr802 target genes yielded by this analysis include key players in multiple processes implicated in cryptococcal virulence, including quorum sensing, Titan cell formation, and stress resistance (Data Set S1, sheets 4 and 5).

### Pdr802 regulates the expression of quorum sensing proteins that control Titan cell formation.

The most striking phenotype we observed in cells lacking *PDR802* is the marked increase in Titan cell formation. We therefore examined our DTO target list for genes known to influence this phenotype, such as those involved in quorum sensing. Recent studies have shown that the quorum sensing peptide Qsp1 is a negative regulator of Titan cell formation (47, 48); Titan cell formation increases upon deletion of the gene encoding this peptide (*QSP1*) or proteins that mediate its maturation and import (*PQP1* and *OPT1*, respectively). We found that Pdr802 positively regulates *PQP1* and *OPT1* gene expression (Table 1), consistent with its repression of Titan cell formation.

**TABLE 1:** Pdr802 target genes involved in quorum sensing and Titan cell formation

Biological process	CNAG ID^*a*^	Gene name	Change determined by:		Description
			ChIP-Seq^*b*^	RNA-Seq^*c*^	
Quorum sensing	00150	*PQP1*	1.38	−0.78	Peptidase
	03013	*OPT1*	1.25	−0.55	OPT small oligopeptide transporter
Titan cell formation	05835	*LIV3*	1.52	−0.84	Transcription factor
	05785	*STB4*	3.33	−1.71	Transcription factor
	05940	*ZFC3*/*CQS2*	2.23	0.68	Transcription factor

a CNAG, ***C**ryptococcus **n**eoformans* serotype **A g**enome project gene identifier (118).

b Fold change for mCherry-Pdr802 compared to WT.

c Log_2_ fold change for *pdr802* compared to WT.

A study of C. neoformans cells exposed to Titan cell-inducing conditions *in vitro* reported that 562 genes were upregulated under this condition, while 421 genes were downregulated (48). The overlap of these genes with our DTO set of Pdr802-regulated genes included the TF genes *LIV3*, *STB4*, and *ZFC3* (48) (Data Set S2, sheets 1 and 2). The first two are repressed during Titan cell induction, while *ZFC3* (also known as *CQS2*) is induced. Our analysis showed that Pdr802 positively regulates expression of *LIV3* and *STB4*, while it negatively regulates *ZFC3* (Table 1), in concordance with our phenotypic observations of Titan cell formation. Notably, Liv3 and Zfc3 are responsive to the peptide Qsp1 (74, 75) and are important for C. neoformans virulence, while Stb4 influences cryptococcal brain infection (67).

### Pdr802 coordinates cryptococcal response to the host environment.

C. neoformans deploys a variety of proteins to resist the many challenges it experiences upon host entry, which include oxidative and temperature stress. Multiple genes that are central to these responses were identified as direct, functional targets of Pdr802 by our DTO analysis (Table 2). For example, Pdr802 induces the expression of genes whose products detoxify reactive oxygen species (ROS), such as *CAT1*, *CAT2*, and *SOD1* (78, 79), or participate in resistance to these compounds, such as *FZC34*, *MIG1*, and *CCK1* (52, 80, 81) (Table 2). Both the kinase Cck1 (also known as Yck2) and the TF Fzc34 have been implicated in cryptococcal virulence (80, 82).

**TABLE 2:** Genes regulated by Pdr802 involved in adaptation to the host environment

Biological process	CNAG ID^*a*^	Gene name	Change determined by:		Description
			ChIP-Seq^*b*^	RNA-Seq^*c*^	
Oxidative stress resistance	04981	*CAT1*	1.47	−1.60	Catalase 1
	05256	*CAT2*	1.41	−0.42	Catalase 2
	01019	*SOD1*	2.59	−0.53	Superoxide dismutase (Cu-Zn)
	00896	*FZC34*	2.22	−0.82	Transcription factor
	06327	*MIG1*	1.72	−0.78	DNA-binding protein CreA
	00556	*CCK1*	1.78	−0.48	Casein kinase I
Melanin and cell wall formation	03202	*CAC1*	1.60	−0.61	Adenylate cyclase
	01845	*PKC1*	2.00	−0.48	AGC/PKC protein kinase
	07724	*CUF1*	1.27	−0.67	Metal-binding regulatory protein
	00740	*SNF5*	1.28	−0.70	Swi/Snf chromatin-remodeling subunit
	01239	*CDA3*	2.63	1.08	Chitin deacetylase 3
	01230	*MP98*	2.74	0.96	Chitin deacetylase 2
	00776	*MP88*	1.86	1.23	Immunoreactive mannoprotein
Growth at 37°C	00405	*KIC1*	1.88	−0.97	Ste/Ste20/Ysk protein kinase
	03670	*IRE1*	1.66	−1.14	IRE protein kinase
Urease activity	07448	*DUR3*	2.13	−3.05	Urea transporter
	01209	*FAB1*	2.29	−0.49	1-Phosphatidylinositol-3-*P* 5-kinase
	01938	*KIN1*	1.61	−0.34	CAMK/CAMKL/KIN1 protein kinase
	01155	*GUT1*	2.96	0.62	Glycerol kinase
	00791	*HLH1*	1.71	−1.09	Transcription factor
Capsule thickness	02802	*ARG2*	1.56	−0.59	Inositol/phosphatidylinositol kinase
	06809	*IKS1*	2.28	−0.44	IKS protein kinase
	01905	*KSP1*	2.02	−0.64	Serine/threonine protein kinase
	02877	*FZC51*	1.75	−0.69	Transcription factor
	07470	*PDE2*	2.42	−1.80	High-affinity phosphodiesterase
	03346	*BZP4*	3.52	1.30	Transcription factor
Calcineurin signaling	00156	*CRZ1*	1.44	−0.49	Transcription factor
	01744	*HAD1*	2.26	0.47	Phosphatase

a CNAG, ***C**ryptococcus **n**eoformans* serotype **A g**enome project gene identifier (118).

b Fold change for mCherry-Pdr802 compared to WT.

c Log_2_ fold change for *pdr802* compared to WT.

As noted above, the pigment melanin has important antioxidant properties that promote cryptococcal survival inside the host (16). Under host-like conditions, Pdr802 regulates genes required for melanization, even though it melanizes normally *in vitro*. These genes include *CAC1*, *PKC1*, *CUF1*, and *SNF5* (Table 2). Cac1 is an adenylyl cyclase responsible for cyclic AMP (cAMP) production in C. neoformans, which plays a central role in melanin synthesis as well as proper capsule production, mating, and virulence (83). The kinase Pkc1 induces production of the laccase (Lac1) that forms melanin and plays a key role in resistance to oxidative and nitrosative stress (84, 85), the TF Cuf1 regulates *LAC1* expression and is important for cryptococcal virulence (86, 87), and *SNF5* is required for full melanization (88). Melanin occurs in the fungal cell wall, which is another key component in fungal stress resistance. Pdr802 is also a direct, functional regulator of several genes whose products influence cell wall glycan content: two chitin deacetylases (Cda3 and Mp98) and the mannoprotein MP88 (Table 2). This is also consistent with the increases in mannose and chitin that occur in Titan cell walls (51).

Pdr802 positively regulates the expression of several proteins required for yeast survival at 37°C, including the kinases Kic1 and Ire1 (Table 2). Ire1 is a regulator of the cryptococcal unfolded-protein response (UPR) pathway and lack of Ire1 or Kic1 impact C. neoformans virulence (80, 89). Pdr802 also modulates cryptococcal urease activity, which is required for dissemination to the central nervous system (CNS) (11, 12), by regulating the urea transporter Dur3 and other proteins that influence urease activity (e.g., the kinases Fab1, Kin1, and Gut1 and the TF Hlh1) (Table 2). Deletion of *FAB1*, *KIN1*, or *HLH1* impaired urease activity in C. neoformans, while *GUT1* disruption induced it (52, 80).

Above, we document the role of Pdr802 in capsule synthesis, which is dramatically upregulated in the host environment in general and is further increased in cells lacking this TF. We found that Pdr802 is a positive regulator of multiple genes that have been implicated in reducing cryptococcal capsule thickness. These include the kinases Arg2, Iks1, and Ksp1; the TF Fzc51; and the phosphodiesterase Pde2 (Table 2). Notably, null mutants for those genes are hypercapsular, similar to *pdr802* cells (52, 80, 90). Pdr802 is a negative regulator of the TF Bzp4, which, as mentioned above, positively regulates capsule (Table 2) (52).

### Pdr802 regulates calcineurin target genes.

**FIG 8:**
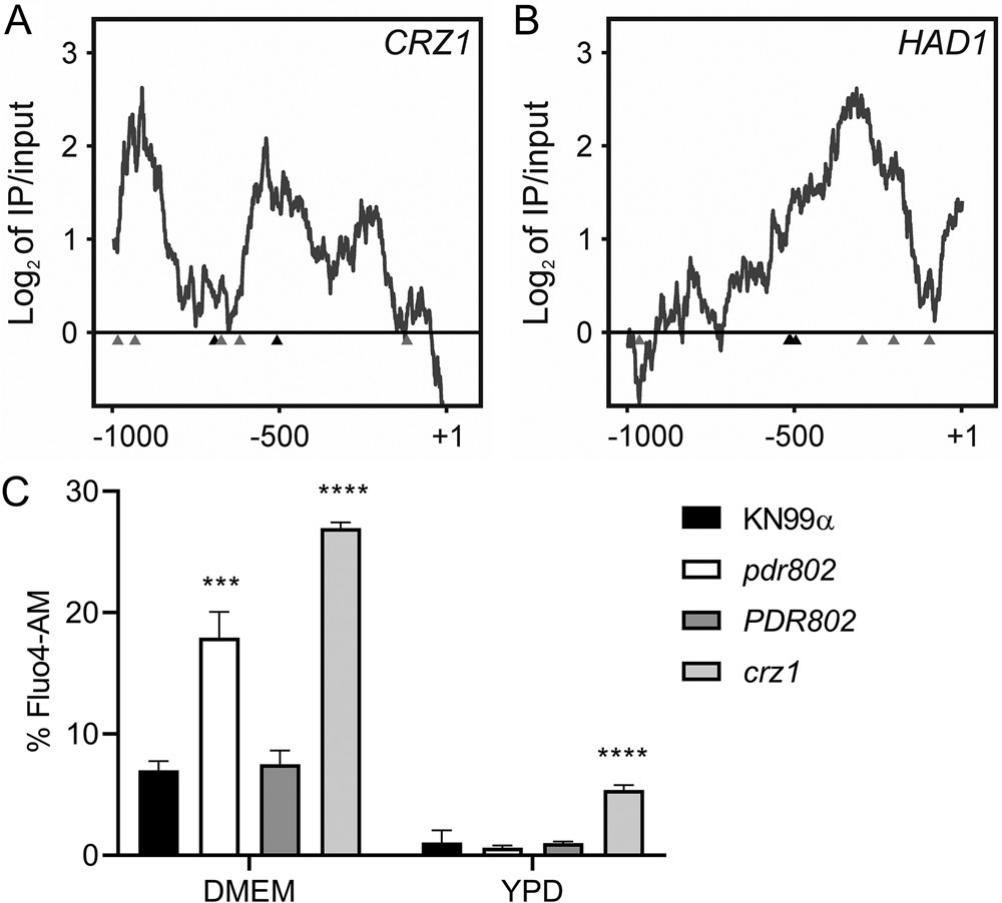
Pdr802 participates in calcineurin signaling. (A and B) Interactions of Pdr802 with upstream regions of the indicated genes. The ratios (log_2_) of reads from immunoprecipitated (IP) DNA to input DNA were calculated for 1,000 bp upstream of the first coding nucleotide (+1); shown is the difference in these values between tagged and untagged strains. Black triangles, complete Pdr802 DNA-binding motifs (Fig. S7C); gray triangles, partial motifs. (C) Intracellular calcium measurement by flow cytometry using Fluo-4AM. Each column shows the mean and standard deviation of three biological replicates. ***, *P* < 0.001, and ****, *P* < 0.0001, compared to KN99α by one-way ANOVA with a *post hoc* Dunnett test.

The calcineurin signaling pathway is activated by calcium and governs stress response and virulence in C. neoformans (91–93). One major mediator of calcineurin signaling is the transcription factor Crz1 mentioned above, which is highly responsive to temperature and influences cryptococcal virulence (57, 62). Upon intracellular calcium influx, calcineurin dephosphorylates Crz1, which then translocates to the nucleus and regulates gene expression (57, 63). We found that Pdr802 binds the *CRZ1* gene promoter and positively regulates its expression (Fig. 8A, Table 2, and Data Set S2, sheet 3). Pdr802 also binds and regulates five other genes whose products are dephosphorylated by calcineurin; these include the phosphatase Had1, which is important for cryptococcal cell wall remodeling and virulence (Fig. 8B, Table 2, and Data Set S2, sheet 3) (63, 94).

10.1128/mBio.03457-20.9 DATA SET S2 Pdr802 target analysis and primers used in this study. Sheet 1, Pdr802 regulated genes that are down-regulated during Titan cell formation *in vitro* (48). Sheet 2, Pdr802 regulated genes that are up-regulated during Titan cell formation *in vitro* (48). Sheet 3, targets regulated by Pdr802 that are also dephosphorylated by calcineurin (63). Sheet 4, the intersection of Pdr802 targets and genes that are Crz1-independent calcineurin targets under conditions thermal stress (55). Sheet 5, primers used in this study. Fold enrichment and adjusted *P* (*q*) values throughout are for the DTO results. Download Data Set S2, XLSX file, 0.02 MB. Copyright © 2021 Reuwsaat et al. 2021 Reuwsaat et al. https://creativecommons.org/licenses/by/4.0/ This content is distributed under the terms of the Creative Commons Attribution 4.0 International license.

Because Crz1 helps maintain normal cryptococcal Ca^2+^ concentrations through the regulation of calcium transporters (57), we wondered about the intracellular calcium levels in *pdr802* cells. We found that indeed, after 24 h of growth in DMEM, the level of cytosolic calcium in the mutant significantly exceeded that of WT or complemented strains (Fig. 8C). It was still, however, below that of a *crz1*-null mutant, supporting the idea that Pdr802 is not the sole regulator of *CRZ1* expression. Notably, *PDR802* deletion had no effect in rich medium (YPD), which reinforces our hypothesis that Pdr802 acts primarily under host-like conditions. To further explore the relationship of Pdr802 and calcineurin, we compared published gene expression profiles of a calcineurin mutant (57) to our DTO data set. Of the 393 genes that are differently expressed in the calcineurin mutant under thermal stress, 26 are regulated by Pdr802 (Data Set S2, sheet 4).

## DISCUSSION

We have shown that Pdr802 is a potent regulator of cryptococcal responses to the host environment. In this context, it influences the formation of capsule and Titan cells as well as cellular responses to temperature and oxidative stress, acts as a downstream effector of calcineurin, and modulates calcium availability. The last function is likely achieved through its positive regulation of the transcription factor Crz1, which in turn modulates the calcium transporters Pmc1 and Vcx1 (57). Since calcium ion is a major second messenger in eukaryotic cells, its accumulation in *pdr802* cells affects multiple processes central to host interactions, including stress responses, cell wall integrity, and capsule size (61, 62, 92, 95, 96).

C. neoformans dissemination to the brain is the main driver of patient mortality (2). We found that dissemination of *pdr802* cells is significantly impaired, although they do occasionally reach the brain. These observations can be explained by a combination of factors. First, the limited accumulation of the *pdr802* mutant in the lungs, due to factors summarized above, may directly affect dissemination (97). Second, this strain survives poorly in mouse serum, as demonstrated directly by our culture experiments and indirectly by our inability to detect it in the blood of infected mice, even 75 days after infection. The latter might occur because the cells do not reach the blood or because they are rapidly eliminated, consistent with previous observations (98). Third, the thick capsules of the *pdr802* mutant reduce its ability to reach the brain. This is true whether fungal entry occurs directly, by the movement of free fungi across the BBB, or indirectly, via a Trojan horse mechanism that requires macrophage uptake (99); such uptake is impeded by enlarged capsules, independent of cell size (31). Fourth, calcium imbalance directly affects cryptococcal transmigration (100). Finally, *pdr802* cells show reduced expression of genes required for urease activity, which promotes C. neoformans dissemination to the CNS (11, 12, 100). Interestingly, despite all of these obstacles to dissemination, mutant cells that do reach the brain are able to proliferate to wild-type levels.

Titan cells are a robust and persistent morphotype of C. neoformans that contribute to yeast virulence (45). We showed that cells lacking Pdr802 demonstrate increased formation of Titan cells *in vivo* and *in vitro*, suggesting that this TF is a novel repressor of this process. Although Titan cells enhance aspects of cryptococcal pathogenesis (19, 101), their overproduction negatively impacts dissemination to the brain due to their resistance to phagocytosis by macrophages (19, 45) and decreased penetration of biological barriers (19).

Our combined analysis of DNA binding and gene expression data allows us to understand the increase in Titan cell formation that occurs upon deletion of *PDR802*. Under host-like conditions, Pdr802 positively regulates Pqp1, Opt1, and Liv3, all key proteins in the cryptococcal quorum sensing pathway, which represses Titan cell formation (47, 48). In the absence of this TF, quorum sensing is impaired, a situation known to increase Titan cell formation. Pdr802 may also indirectly regulate Titan cell formation by regulating other TFs that impact this process, such as Zfc3 (Cqs2) and Stb4.

We know that capsule, a key virulence factor, is typically highly induced in the host or under host-like conditions (102). Our studies *in vitro*, *ex vivo*, and *in vivo* show that Pdr802 normally reins in this process. This likely occurs via a combination of Pdr802’s repression of the TF Bzp4, which positively regulates capsule size, and the induction of other factors (e.g., the TF Fzc1, the phosphodiesterase Pde2, and the kinases Ksp1, Arg2, and Iks1) that negatively regulate capsule size (52, 80, 90).

**FIG 9:**
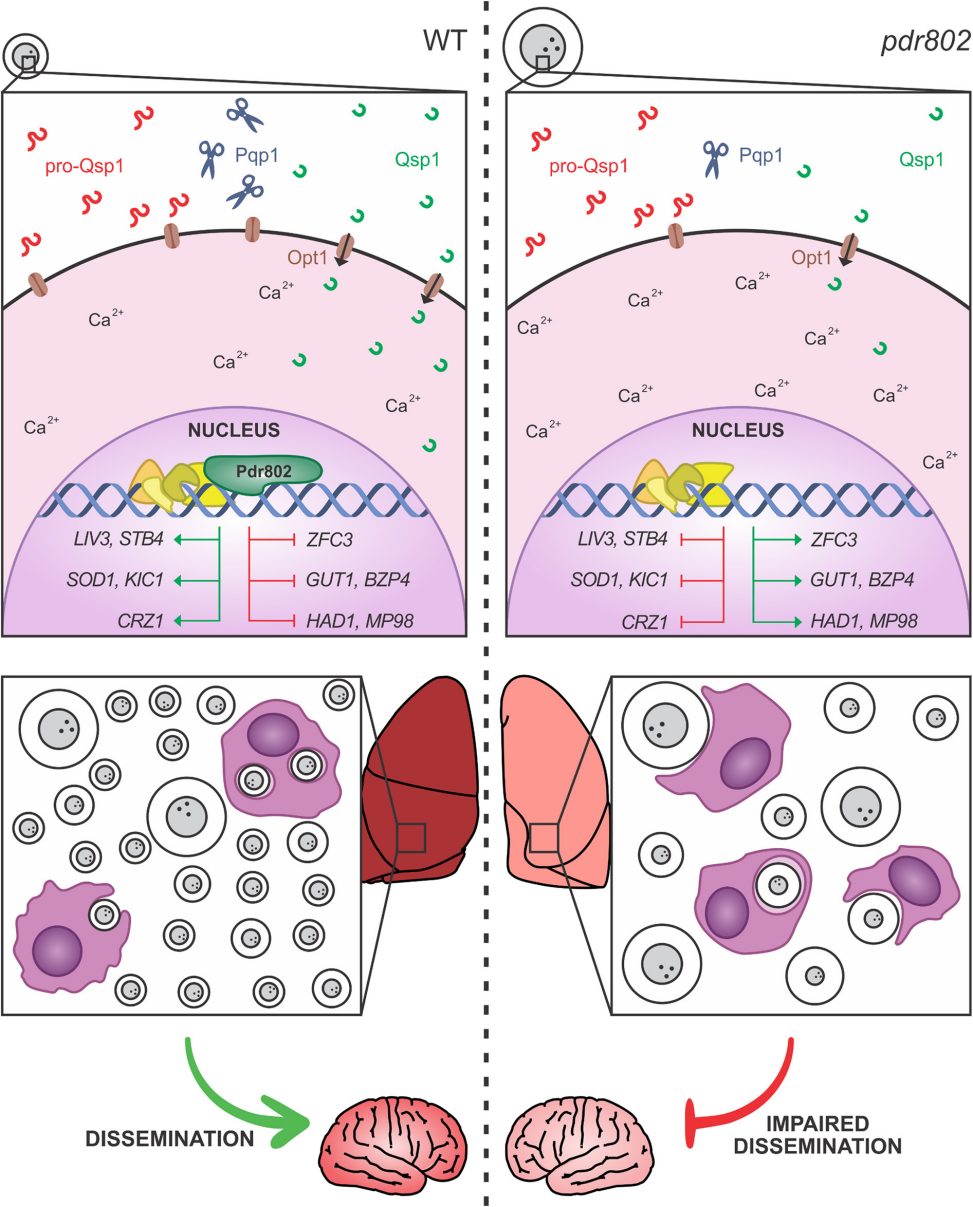
Pdr802 mode of action. (Left) When wild-type C. neoformans enters a host, *PDR802* expression is induced and Pdr802 positively regulates elements of the quorum sensing pathway (described in the text) as well as expression of TFs implicated in this pathway (*LIV3*), Titan cell production (*ZFC3*), and brain infectivity (*STB4*). At the same time, Pdr802 regulates two calcineurin targets (*CRZ1* and *HAD1*) and a variety of other genes (see the text). Shown are a few examples of genes involved in the response to oxidative stress (*SOD1*), growth at 37°C (*KIC1*), urease activity (*GUT1*), capsule production (*BZP4*), and cell wall remodeling (*MP98*). (Right) In the absence of these regulatory changes, *pdr802* cells are poorly equipped to survive the stress of the host environment and are subject to increased intracellular calcium levels, dysregulation of capsule production, and impaired stress resistance. As a result, the cryptococcal population in the lung is smaller and is enriched in Titan cells and hypercapsular cells of normal size, both of which demonstrate reduced phagocytosis by host cells and impaired ability to cross biological barriers; these defects reduce dissemination to the central nervous system.

Overall, we found that Pdr802 influences key cryptococcal phenotypes that influence virulence, including quorum sensing, stress responses, Titan cell formation, and capsule production (Fig. 9). We have further identified multiple genes that are central in these processes and are directly regulated by Pdr802. Some of these targets are also regulated by calcineurin (e.g., Had1 and Crz1) or by another important TF, Gat201 (e.g., Opt1, Liv3, and Zfc3) (60, 74, 75). Finally, the expression of *PDR802* itself is regulated by the TFs Gat201 and Hob1 (67, 75). The cross talk between all of these regulatory mechanisms remains to be dissected. Nonetheless, it is evident that Pdr802 is critical for both survival in the lung and dissemination to the brain, thus explaining its role in cryptococcal virulence.

## MATERIALS AND METHODS

### Strain construction and cell growth.

We previously reported the *PDR802* deletion mutant (*pdr802*) in the KN99α strain background (103) that was used in this work (39). Complementation of this mutant with the wild-type gene at the native locus (*PDR802*) and construction of a strain that expresses Pdr802 with N-terminal mCherry (mCherry-Pdr802) are detailed in the supplemental methods. For all studies, C. neoformans strains were inoculated from single colonies into YPD medium (2% [wt/vol] dextrose, 2% [wt/vol] Bacto peptone, and 1% [wt/vol] yeast extract in double-distilled water [ddH_2_O]) and grown overnight at 30°C with shaking at 230 rpm before further handling as detailed below. To assess viability during growth in tissue culture medium, overnight cultures were washed with phosphate-buffered saline (PBS), diluted to 10^6^ cells/ml in DMEM (Sigma; D6429), plated (1 ml/well) in triplicate in 24-well plates, and incubated at 37°C and 5% CO_2_. At the indicated times, cells were mixed thoroughly, diluted in PBS, and plated on YPD agar (YPD medium, 2% agar [wt/vol]) for assessment of CFU. To assess viability during growth in mouse serum, mouse blood was collected as described below in “Animal experiments”; YPD-grown cryptococcal cells were incubated in 100 μl of serum in 96-well plates for 24 h at 37°C and 5% CO_2_, and CFU were counted as described above.

### Animal experiments.

All animal protocols were approved by the Washington University Institutional Animal Care and Use Committee (reference 20170131) or Comissão de Ética no Uso de Animais (CEUA) (reference 30936), and care was taken to minimize handling and discomfort. For survival studies, groups of five 4- to 6-week-old female C57BL/6 mice (The Jackson Laboratory) were anesthetized by subcutaneous injection of 1.20 mg ketamine and 0.24 mg xylazine in 120 μl sterile water and intranasally infected with 5 × 10^4^ cryptococcal cells. The mice were monitored and humanely sacrificed when their weight decreased to below 80% of initial weight or if they showed signs of disease, at which point organ burden was assessed. The lungs and brains were harvested, homogenized, diluted, and plated on YPD agar. The resulting CFU were enumerated, and survival differences were assessed by Kaplan-Meier analysis.

For timed organ burden studies, C. neoformans overnight cultures were centrifuged (1,000 × *g* for 3 min), washed with sterile PBS, and resuspended in PBS to 1 × 10^6^ cells/ml. Groups of three 4- to 6-week-old female C57BL/6 mice (Centro Multidisciplinar para Investigação Biológica na Área da Ciência em Animais de Laboratório [CEMIB]) were anesthetized as described above, intranasally infected with 5 × 10^4^ cryptococcal cells, and monitored as described above. At set time points post-infection (see above), mice were sacrificed and fungal burden was assessed from organs (as described above) or blood (obtained by cardiac puncture). Organ burden was analyzed by Kruskal-Wallis test with Dunn’s multiple comparison *post hoc* test for each day post-infection.

To assess cryptococcal viability in mouse serum, 6 BALB/c mice were anesthetized with isoflurane and blood was collected from the retro-orbital space using a sterile capillary tube. Collected blood was incubated at 37°C for 30 min, and serum was isolated by centrifugation at 1,000 × *g* for 15 min and then heat inactivated at 56°C for 30 min.

### Capsule analysis.

To qualitatively assess capsule thickness, strains were grown on YPD medium for 16 h and washed with PBS, and 10^3^ cells were incubated in mouse serum for 24 h at 37°C and 5% CO_2_. After incubation, cells were fixed in 4% paraformaldehyde and washed three times with PBS. C. neoformans cells were placed on glass slides and mixed with similar volumes of India ink, and the capsule was measured as previously described (104).

For population-level capsule measurement, C. neoformans strains were grown overnight in YPD, washed with PBS, and diluted to 10^6^ cells/ml in DMEM. Aliquots (150 μl) were then plated in quadruplicate in the middle 32 wells of a poly-l-lysine-coated 96-well plate (Fisher; 655936) and incubated at 37°C and 5% CO_2_. After 24 h, the cells were washed with PBS and incubated with 150 μl of a staining mixture (100 μg/ml calcofluor white to stain cell walls, 50 μg/ml of the anticapsular monoclonal antibody 302 conjugated to Alexa Fluor 488 [Molecular Probes], and 1.5% goat serum in PBS) for 30 min at room temperature in the dark. The cells were washed again with PBS, fixed with 4% formaldehyde for 10 min at room temperature, and washed with PBS, and each well was refilled with 150 μl PBS. The cells were imaged using a BioTek Cytation 3 imager, which automatically collected 100 images per well in a grid pattern at the well center. Image files were prepared for analysis with GE Healthcare IN Cell Translator and assembled into .xdce image stacks for analysis with GE Healthcare IN Cell Developer Toolbox 1.9. Cell wall and capsule images were first filtered to remove background noise and border objects, and then cells were identified using shape-based object segmentation (3-pixel kernel, 50% sensitivity) followed by watershed clump breaking to prevent apparent connectivity caused by incomplete segmentation. Target linking was performed to assign each cell wall object to one capsule object based on known 1:1 pairing and location, generating a target set. Capsule and cell wall object diameters were calculated for each target set (hundreds to thousands per well), and the difference between measurements in each pair was defined as the capsule thickness. Data were normalized by the difference in capsule thickness between uninduced and induced WT cells, which were included in each experiment, and compared to data for hypercapsular (*pkr1*) (39) and hypocapsular (*ada2*) (105) control strains in each experiment. Capsule sizes were compared by one-way analysis of variance (ANOVA) with Dunnett’s multiple comparison *post hoc* test.

To measure capsule thickness of cryptococcal cells grown in the lungs of infected mice, lung homogenates were filtered through a cell strainer with 40-μm pores using a syringe plunger, fixed in 3.7% formaldehyde, and used for India ink staining and measurement as described above. For the visualization of KN99α and *PDR802* cells from mouse lungs after 18 days of infection, the tissue was treated with 50 μg/ml DNase I for 30 min at 37°C.

GXM immunoblotting was conducted as previously described (71). Briefly, 10^6^ cells/ml were grown in DMEM for 24 and 48 h. Culture supernatant fractions were then resolved by gel electrophoresis on 0.6% agarose, transferred onto nylon membranes, and probed with 1 μg/ml anti-GXM antibody 302.

### Phenotypic assays.

For stress plates, cryptococcal cells were grown overnight in YPD, washed with PBS, and diluted to 10^7^ cells/ml in PBS. Aliquots (3 μl) of 10-fold serial dilutions were spotted on YPD or YNB agar supplemented with various stressors (sorbitol, NaCl, CaCl_2_, LiCl, Congo red, calcofluor white, caffeine, SDS, NaNO_2_, H_2_O_2_, and ethanol) at the concentrations indicated in the figures. Melanization was tested on plates made by mixing 10 ml of 2× minimal medium (2 g/liter l-asparagine, 1 g/liter MgSO_4_ ⋅ 7H_2_O, 6 g/liter KH_2_PO_4_, 2 g/liter thiamine, 2 mM l-3,4-dihydroxyphenylalanine [l-DOPA]; 0.1% dextrose was added for melanization induction, and 0.5% was added for melanization inhibition) with 10 ml of 2% agar-water per plate. A control strain lacking the ability to melanize was used as a control (*lac1*) (88). For the solid urease assay, 10 μl of a 10^7^ cells/ml suspension in water was plated on Christensen’s urea solid medium (1 g/liter peptone, 1 g/liter dextrose, 5 g/liter NaCl, 0.8 g/liter KH_2_PO_4_, 1.2 g/liter Na_2_HPO_4_, 0.012 g/liter phenol red and 15 g/liter agar; pH 6.8). Plates were incubated at 30°C or 37°C.

### Titan cells.

Titan cell induction was performed in 1× PBS supplemented with 10% heat-inactivated fetal calf serum (FCS) for 72 h at 37°C and 5% CO_2_ as recently described (49) and quantified by flow cytometry as previously reported (47, 48). For analysis of DNA content, cells were treated as previously described (47) with a few modifications. Briefly, cultures were fixed with 70% ethanol for 1 h at 24°C and then overnight at 4°C, washed twice with PBS, and then treated with propidium iodide (50 μg/ml) and RNase A (0.5 mg/ml) in PBS for 2 h at 30°C with agitation. Quantification was performed using a FACS ARIA III (BD) cytometer with analysis on the Cytobank platform. A total of 20,000 events were recorded, and doublets were filtered out using comparisons of forward-scatter width (FSC-W) versus forward-scatter height (FSC-H) to exclude events with high FSC-W and FSC-H. To define the 1C and 2C gates, cells were grown in yeast nitrogen base (YNB) (24 h, 30°C, 150 rpm) without or with 80 μg/ml benomyl, which traps cells in 2C, as recently reported (106).

### Phagocytosis.

J774.16 cells were prepared for uptake experiments by seeding (10^5^ cells/well) in a 96-well plate and incubating in DMEM supplemented with 10% fetal bovine serum (FBS) at 37°C and 5% CO_2_ for 24 h. C. neoformans cells were prepared for uptake experiments by inoculating an overnight culture in YPD into either DMEM or Titan cell induction medium (49) and growing at 37°C and 5% CO_2_ for 24 or 72 h, respectively. To initiate the study, cryptococcal cells were washed with PBS and opsonized with anti-capsular antibody 18B7 (1 μg/ml) for 1 h at 37°C, while macrophages were activated with 50 nM phorbol myristate acetate (PMA) for 1 h at 37°C and 5% CO_2_; 10^6^ cryptococcal cells were then incubated with the macrophages for 2 h at 37°C and 5% CO_2_. The wells were then washed three times with warm PBS, and the macrophages were lysed with 0.1% Triton in PBS and plated for CFU enumeration as described above. Fold change in numbers of CFU was assessed by comparison to the CFU of opsonized cells. One-way ANOVA with Dunnett’s multiple-comparison *post hoc* test was used to compare phagocytosis of *pdr802* and *PDR802* strains with that of KN99α.

### Chromatin immunoprecipitation.

ChIP studies were performed as previously described (39, 105). Briefly, wild type and N-terminal-mCherry-Pdr802 strains were cultivated in DMEM for 24 h at 37°C and 5% CO_2_. The cells were then fixed with formaldehyde and lysed by mechanical bead-beating, and the cell debris was removed by centrifugation. The supernatant fraction was sheared by sonication and centrifuged, and an aliquot was reserved as “input.” The remaining material was incubated with rabbit IgG anti-mCherry antibody (Abcam; ab213511) tethered to protein A Sepharose (IP) or Sepharose alone overnight at 4°C. The beads were then washed, incubated at 65°C to reverse DNA-DNA and DNA-protein cross-links, and the DNA was recovered by phenol-chloroform-isoamyl alcohol (25:24:1) extraction, ethanol precipitation, and resuspension in nuclease-free water.

Samples were submitted to the Washington University Genome Technology Access Center for library preparation, and DNA samples were sequenced using the Illumina NextSeq platform. The first replicate was sequenced using paired-end 2 × 75-bp reads, and replicates 2 and 3 were sequenced using single-end 75-bp reads; the minimum coverage obtained was ∼16×. The quality of the reads was evaluated by FastQC (107). Fastq files were aligned to the KN99 genome (108) using NextGenMap 0.5.3 (109). SAM files were converted to bam, reads were sorted and indexed, and read duplicates were removed from the final bam files using SAMtools (110). SAMtools was also used to filter out reads with a mapping quality of less than 20 phreds to guarantee single alignment of the reads. Peaks were called using MACS2 (2.1.1.20160309) (111), filtered by size (maximum threshold of 5 kb and no minimum), and annotated using Homer 4.8 (112). The significant peaks were chosen using the cutoff of fold enrichment above 2 and an adjusted *P* value of <0.05, and read coverage of each peak was obtained using SAMtools (110). Pdr802 binding motifs were identified using DREME (76); partial motifs were defined as at least 5 consecutive base pairs of the motif.

### RNA-Seq and DTO.

RNA from wild-type and *pdr802* cells grown for 24 h in DMEM (37°C, 5% CO_2_) was isolated and sequenced as previously described (39). Briefly, cDNA samples were sequenced using the Illumina NextSeq platform for paired-end 2 × 75 bp reads and read quality was evaluated by FastQC (107). Fastq files were aligned to the KN99 genome (108) using NovoAlign (113), SAM files were converted to bam, reads were sorted and indexed, and read duplicates were removed from the final bam files using SAMtools (110). The number of reads mapped per gene was calculated using HTSeq (114), and differential gene expression was analyzed with DESeq2 (115), using the independent hypothesis weighting (IHW) package to calculate the adjusted *P* values (116). Dual-threshold optimization (DTO) analysis was performed as recently described (77). This is a method for simultaneously finding the best thresholds for significance in a TF binding location data set (e.g., ChIP) and a TF perturbation response data set (e.g., RNA-Seq of a TF mutant). It works by trying out all pairs of thresholds for the two data sets, picking the pair that minimizes the probability of the overlap between the bound and responsive gene sets occurring by chance under a null model, and testing the significance of the overlap by comparison to randomly permuted data. Our application of DTO to our ChIP and RNA-Seq data yielded 1,455 bound genes, 5,186 responsive genes, and 1,167 genes that were both bound and responsive. Based on DTO, Pdr802 has an acceptable convergence from binding and perturbation, with a *P* value of <0.01 from the random permutation test and minimum expected false discovery rate (FDR) less than or equal to 20% at 80% sensitivity. In addition to requiring a statistically significant overlap between the ChIP-Seq and RNA-Seq gene sets, we filtered out any genes for which traditional differential expression analysis yielded an adjusted *P* value of ≤0.15 or absolute fold change of ≥0.3, leaving 380 bound targets.

### Intracellular calcium measurement.

To measure intracellular free Ca^2+^, yeast cells were cultured overnight in YPD at 30°C with shaking, washed three times with deionized water, diluted to 10^6^ cells/ml in DMEM (Sigma; D6429), plated (1 ml/well) in triplicate in 24-well plates, and incubated at 37°C and 5% CO_2_ for 24 h. At the indicated times, cells were mixed thoroughly, diluted in PBS containing 2 μM Fluo4-AM (Thermo Fisher), incubated at 30°C for 30 min, and analyzed using flow cytometry. The overnight culture was used as a control and treated as above.

### Data availability.

ChIP-Seq and RNA-Seq data files are available at the NCBI Gene Expression Omnibus under accession numbers GSE153134 and GSE162851, respectively.

10.1128/mBio.03457-20.10 TEXT S1 Supplemental methods. Download Text S1, PDF file, 0.04 MB. Copyright © 2021 Reuwsaat et al. 2021 Reuwsaat et al. https://creativecommons.org/licenses/by/4.0/ This content is distributed under the terms of the Creative Commons Attribution 4.0 International license.
