# Supplementary material for: The Transcription Factor Pdr802 Regulates Titan Cell Formation and Pathogenicity of Cryptococcus neoformans
Source: mBio. 2021 Mar 9;12(2):e03457-20. doi: 10.1128/mBio.03457-20 (PMC8092302; doi:10.1128/mBio.03457-20)
Supplement: FIG S1 [file mBio.03457-20-sf001.pdf]

FIGURE S1

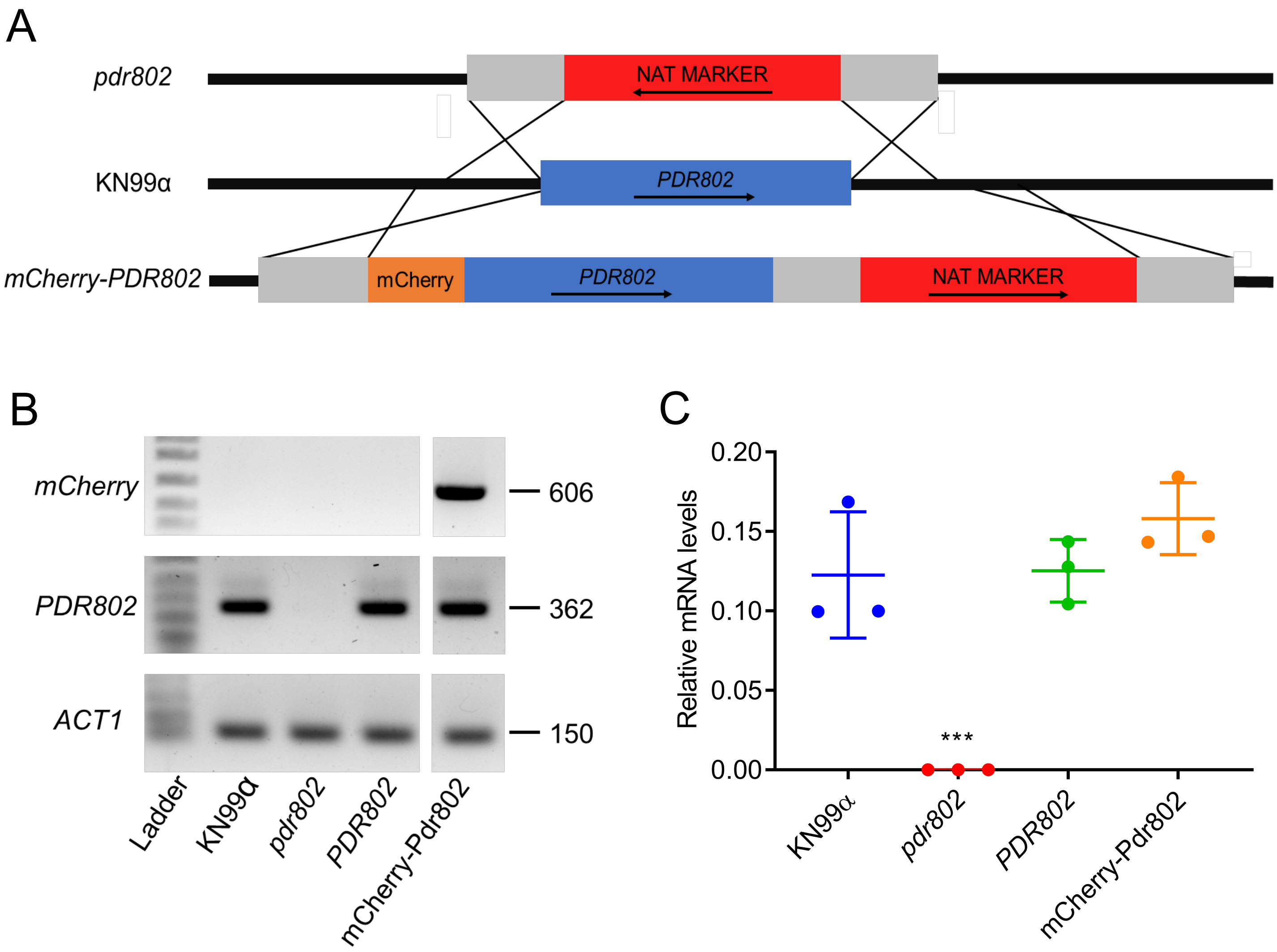

Figure S1. Mutant strain construction and confirmation. A. Scheme for generating *C. neoformans* strains in the KN99α background (middle) that either lack *PDR802* (*pdr802,* top) or encode a tagged copy of the protein (*mCherry-PDR802*). B. Qualitative analysis of gene expression in Panel A strains and the complemented *pdr802* mutant (*PDR802*). Cryptococcal mRNA isolated from cells grown in DMEM (37°C, 5% CO_2, 24 hours) was used to generate cDNA; from this, segments of the genes indicated at the left were amplified using the primers listed in Data Set S2, Sheet 5, and the products were analyzed by agarose gel electrophoresis. Fragment sizes (in bp) are indicated at right and the ladder bands shown are 400, 500, 650, 850 and 1000 bp for the top panel; 200, 300, 400, 500, and 650 bp for the middle panel; and 100, 200, and 300 bp for the bottom panel. C. Quantitative analysis of *PDR802* expression. Samples of RNA isolated as in B were analyzed for *PDR802* expression by qRT-PCR. All results were normalized to *ACT1* expression. Each symbol represents a biological replicate, with the mean and standard deviation also shown. ***, $p<0.001$ compared to KN99α by one-way ANOVA with posthoc Dunnett test.
