# Supplementary material for: The Transcription Factor Pdr802 Regulates Titan Cell Formation and Pathogenicity of Cryptococcus neoformans
Source: mBio. 2021 Mar 9;12(2):e03457-20. doi: 10.1128/mBio.03457-20 (PMC8092302; doi:10.1128/mBio.03457-20)
Supplement: FIG S2 [file mBio.03457-20-sf002.pdf]

FIGURE S2

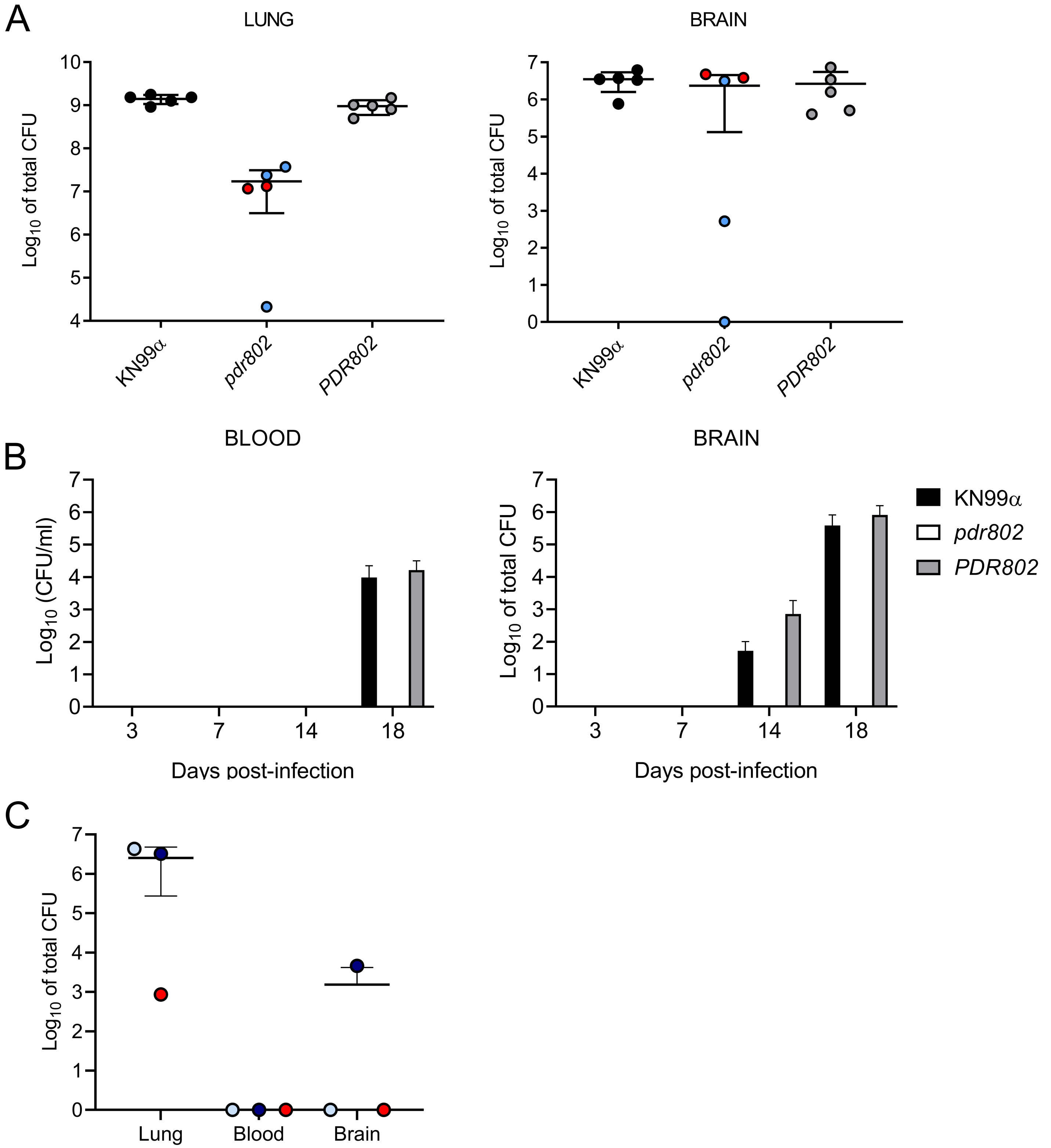

Figure S2. Organ burdens. A. Mean +/- SD values of total colony-forming units (CFU) in the indicated tissue of mice from the Figure 1 survival curve are shown. Each point shows the average value for a single animal at the time of death. For *pdr802* infections, red circles represent mice sacrificed at days 65 and 69, while blue circles represent mice sacrificed at the end of the study (day 100). B. Mean +/- SD of total colony-forming units (CFU) in the blood and brain at the indicated times post-infection. C. Mean +/- SD of total colony-forming units (CFU) in the lung, blood and brain 75 days after infection with *pdr802*. Each color represents one mouse.
