# Supplementary material for: The Transcription Factor Pdr802 Regulates Titan Cell Formation and Pathogenicity of Cryptococcus neoformans
Source: mBio. 2021 Mar 9;12(2):e03457-20. doi: 10.1128/mBio.03457-20 (PMC8092302; doi:10.1128/mBio.03457-20)
Supplement: FIG S3 [file mBio.03457-20-sf003.pdf]

FIGURE S3

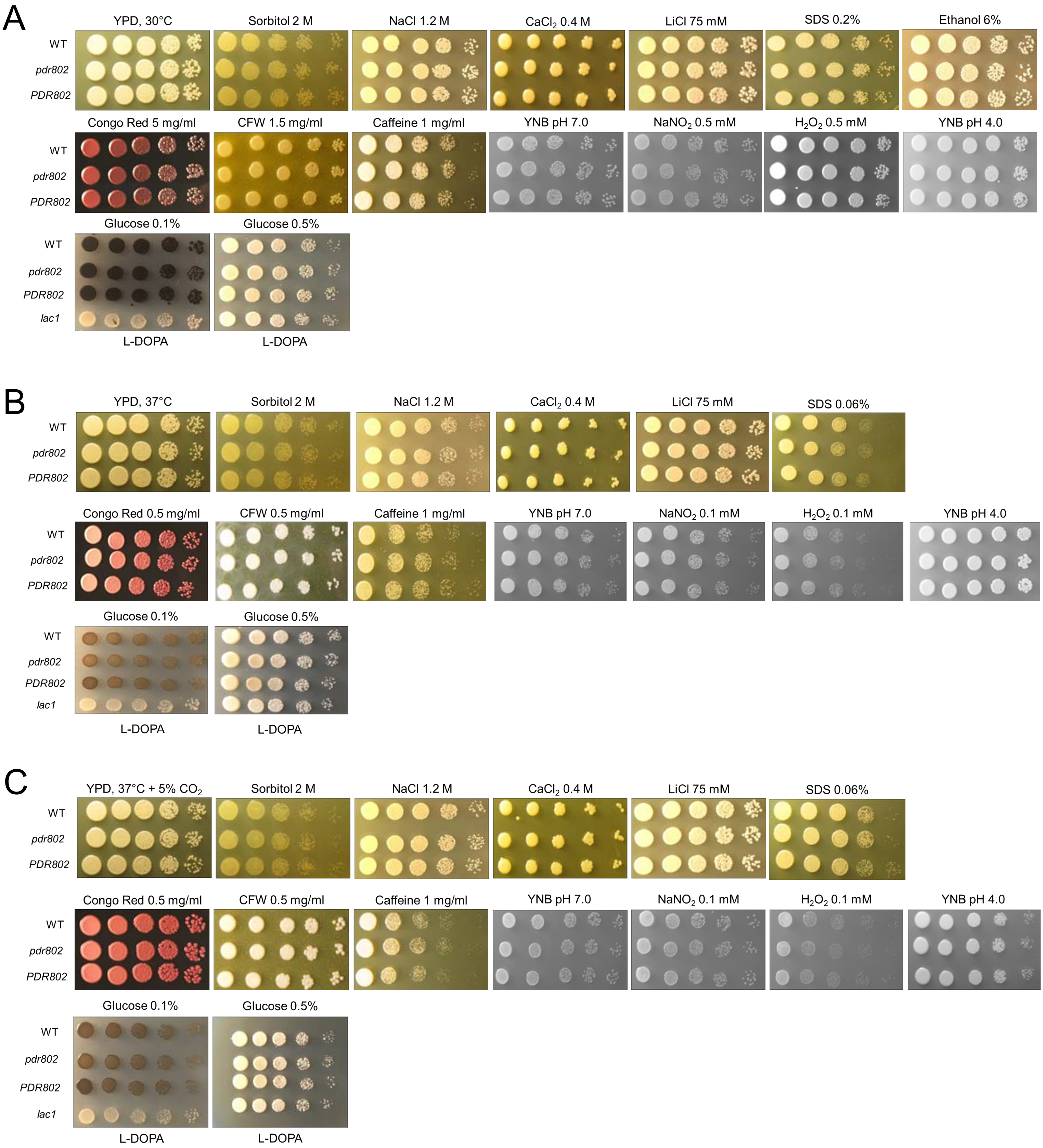
A
YPD, 30°C
Sorbitol 2 M
NaCl 1.2 M
$CaCl_2$ 0.4 M
LiCl 75 mM
SDS 0.2%
Ethanol 6%
WT
pdr802
PDR802
Congo Red 5 mg/ml
CFW 1.5 mg/ml
Caffeine 1 mg/ml
YNB pH 7.0
$NaNO_2$ 0.5 mM
H_2O_2 0.5 mM
YNB pH 4.0
Glucose 0.1%
Glucose 0.5%
lac1
L-DOPA
L-DOPA
B
YPD, 37°C
Sorbitol 2 M
NaCl 1.2 M
$CaCl_2$ 0.4 M
LiCl 75 mM
SDS 0.06%
Congo Red 0.5 mg/ml
CFW 0.5 mg/ml
Caffeine 1 mg/ml
YNB pH 7.0
$NaNO_2$ 0.1 mM
H_2O_2 0.1 mM
YNB pH 4.0
Glucose 0.1%
Glucose 0.5%
L-DOPA
L-DOPA
C
YPD, 37°C + 5% CO_2
Sorbitol 2 M
NaCl 1.2 M
$CaCl_2$ 0.4 M
LiCl 75 mM
SDS 0.06%
Congo Red 0.5 mg/ml
CFW 0.5 mg/ml
Caffeine 1 mg/ml
YNB pH 7.0
$NaNO_2$ 0.1 mM
H_2O_2 0.1 mM
YNB pH 4.0
Glucose 0.1%
Glucose 0.5%
L-DOPA
L-DOPA

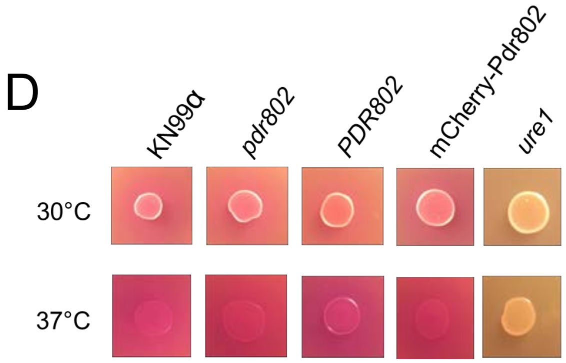

Figure S3. Characterization of *pdr802* cells. *Panels A-C*.10-fold serial dilutions of WT, *pdr802*, and *PDR802* cells were plated on the media shown and incubated at 30°C (A), 37°C (B), or 37°C in the presence of 5% CO_2 (C). Nitrosative ($NaNO_2$) and oxidative (H_2O_2) stress plates were prepared with YNB medium and melanization plates containing L-DOPA were prepared as in the Methods; all other plates were prepared with YPD medium. *lac1*, a control strain lacking the ability to melanize (88). D. Urease activity of the indicated strains was evaluated using Christensen's urea solid medium (see Methods) at the indicated temperatures. *ure1*, a control strain that does not produce urease (17).
