# Supplementary material for: The Transcription Factor Pdr802 Regulates Titan Cell Formation and Pathogenicity of Cryptococcus neoformans
Source: mBio. 2021 Mar 9;12(2):e03457-20. doi: 10.1128/mBio.03457-20 (PMC8092302; doi:10.1128/mBio.03457-20)
Supplement: FIG S4 [file mBio.03457-20-sf004.pdf]

FIGURE S4

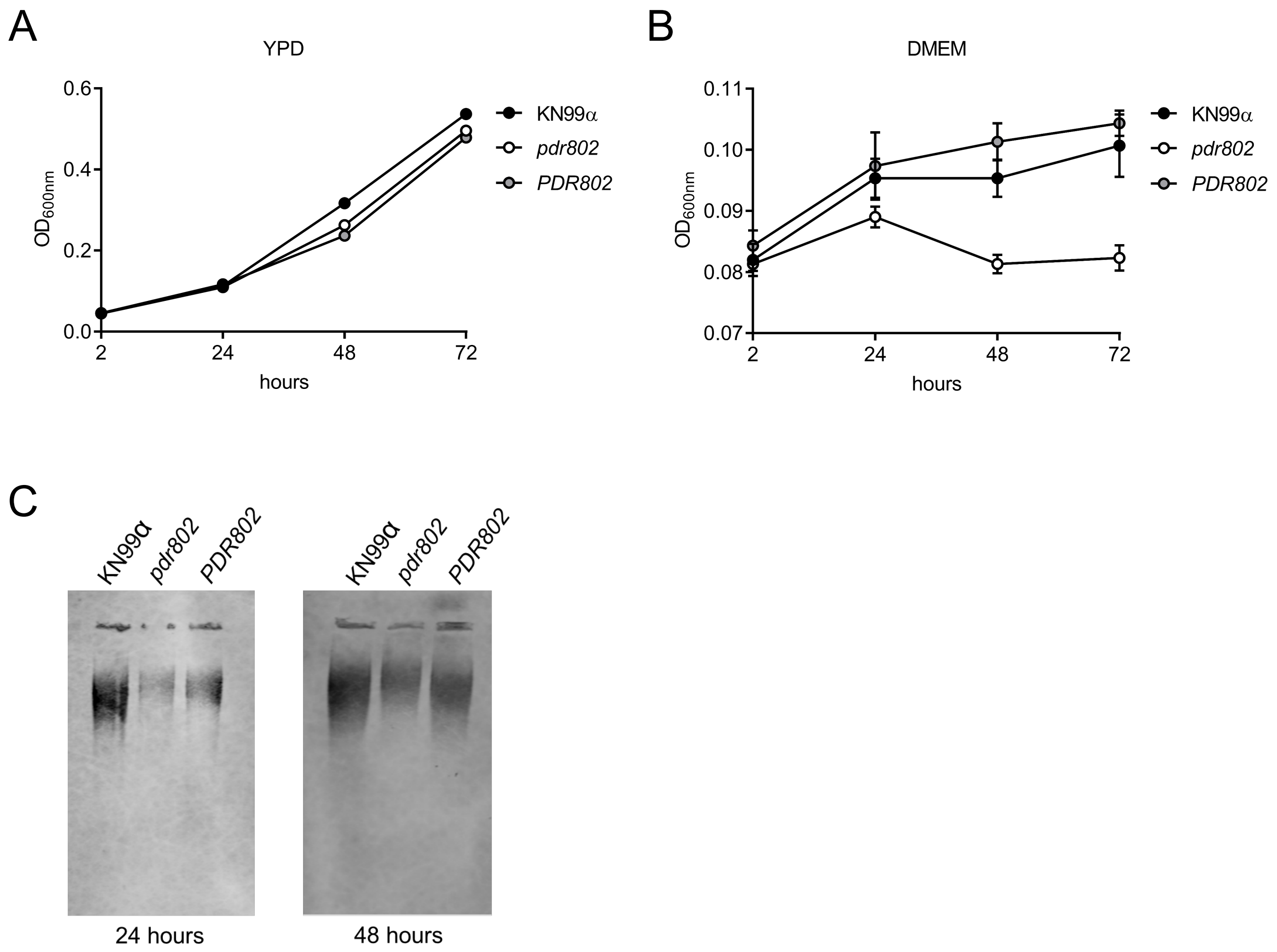

Figure S4. Growth curves and capsule shedding. *Panels A-B*. Growth of the indicated strains in YPD at 30°C (A) or DMEM at 37°C and 5% CO_2 (B) was assessed by OD_{600nm} at the times shown. C. Conditioned medium from the indicated strains was probed for the presence of GXM after growth in DMEM for 24 or 48 hours. Equal volumes of culture supernatant were analyzed without normalization to cell density. Immunoblotting was performed using the anti-GXM monoclonal antibody 302.
