# Supplementary material for: The Transcription Factor Pdr802 Regulates Titan Cell Formation and Pathogenicity of Cryptococcus neoformans
Source: mBio. 2021 Mar 9;12(2):e03457-20. doi: 10.1128/mBio.03457-20 (PMC8092302; doi:10.1128/mBio.03457-20)
Supplement: FIG S5 [file mBio.03457-20-sf005.pdf]

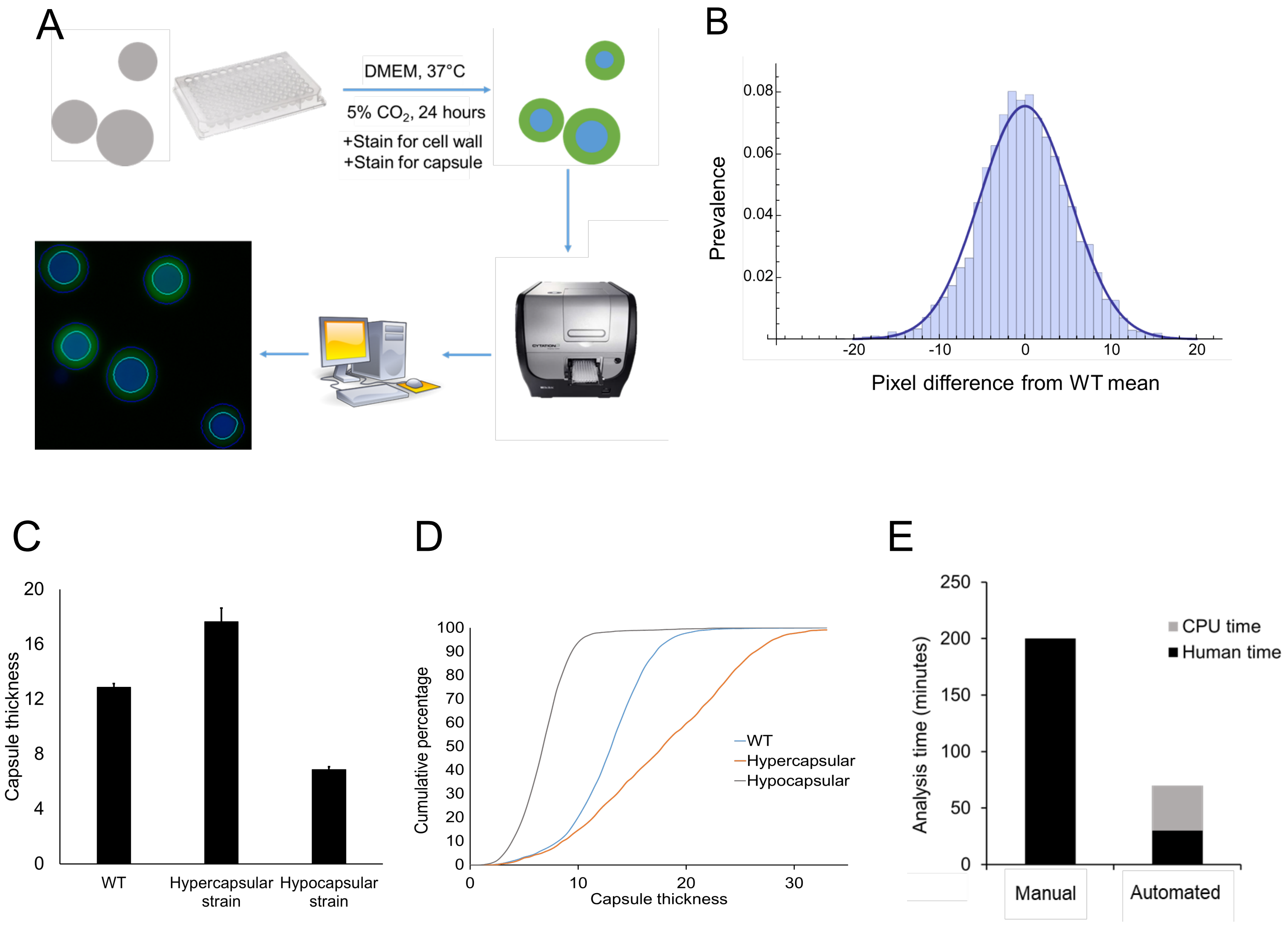

Figure S5. Semi-automated assay for cryptococcal capsule imaging. A. Schematic of applying this method to cryptococcal cells induced to form capsule by growth in DMEM (37°C, 5% CO_2) for 24 h, followed by cell wall and capsule staining. Thousands of cells may be imaged per well and analyzed automatically with software that annotates and measures the capsule (annotated on the micrograph in blue) and cell wall (annotated on the micrograph in bright green). See Methods for details. B. Capsule size distribution of WT cells after induction. Capsule thickness for each cell is the difference between the paired diameters of the cell wall and capsule, which is plotted here with reference to the mean value. C and D. Mean and SD (C) and cumulative percentage (D) analysis of WT compared to hyper and hypocapsular control strains (here *pkr1* and *ada2*, respectively). Capsule thickness is in arbitrary units, related to the pixels measured. E. The time required to analyze the capsule thickness of 1,000 cells by this method compared to manual assessment of India ink images.
