# Supplementary material for: The Transcription Factor Pdr802 Regulates Titan Cell Formation and Pathogenicity of Cryptococcus neoformans
Source: mBio. 2021 Mar 9;12(2):e03457-20. doi: 10.1128/mBio.03457-20 (PMC8092302; doi:10.1128/mBio.03457-20)
Supplement: FIG S6 [file mBio.03457-20-sf006.pdf]

FIGURE S6

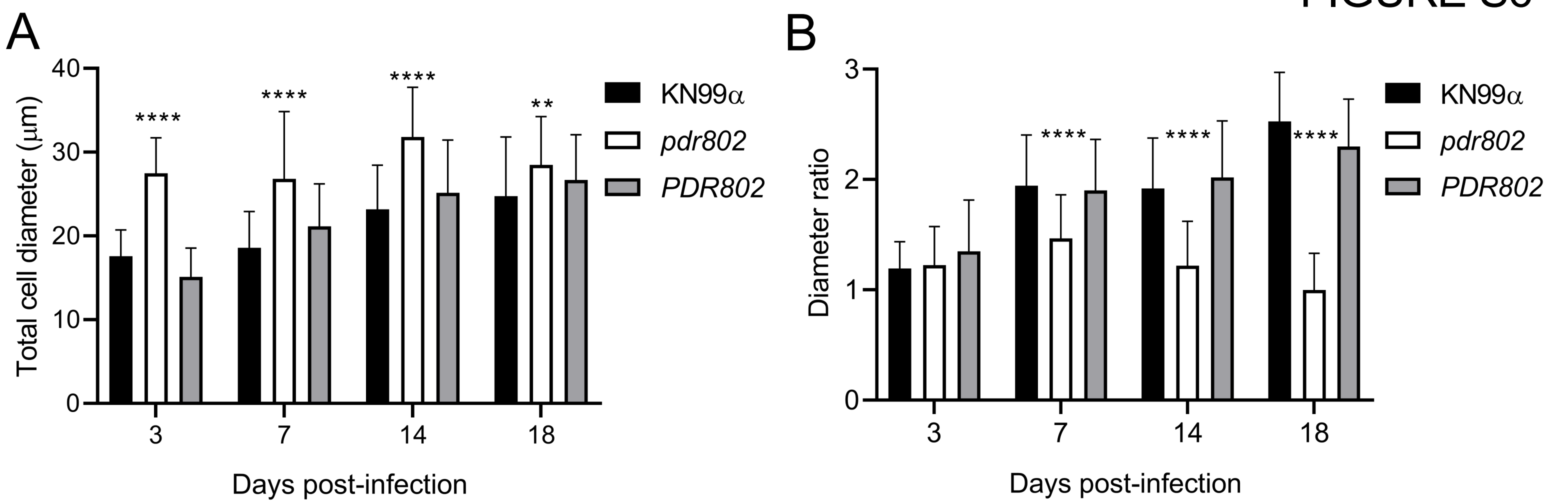

C

3 DPI	KN99α	*pdr802*	*PDR802*
Cell body diameter ≥ 10 µm	20	86.7	11.1
Cell body diameter ≥ 15 µm	0	22.2	0
Total cell diameter ≥ 30 µm	0	31.1	0
**7 DPI**	**KN99α**	***pdr802***	***PDR802***
Cell body diameter ≥ 10 µm	8.5	57.4	8.5
Cell body diameter ≥ 15 µm	0	19.1	2.1
Total cell diameter ≥ 30 µm	0	31.9	2.1
**14 DPI**	**KN99α**	***pdr802***	***PDR802***
Cell body diameter ≥ 10 µm	27.1	87.1	24.3
Cell body diameter ≥ 15 µm	2.9	42.9	4.3
Total cell diameter ≥ 30 µm	12.9	57.1	25.7
**18 DPI**	**KN99α**	***pdr802***	***PDR802***
Cell body diameter ≥ 10 µm	9.1	89.4	15.1
Cell body diameter ≥ 15 µm	0	53.0	1.5
Total cell diameter ≥ 30 µm	21.2	43.9	27.3

Figure S6. *PDR802* deletion induces Titan cell formation. Mean +/- SD of (A) total cell diameter and (B) the ratio of total cell to cell body diameters (diameter ratio), assessed by measuring at least 50 cells per strain with ImageJ. **, $p<0.01$ and ****, $p<0.0001$ for comparison of *pdr802* to KN99α or *PDR802* by one-way ANOVA with posthoc Dunnett test for each day post-infection. C. Percent of Titan cells in the indicated strain, evaluated using various published parameters: cell body diameter above 10 or 15 µm (20) or total cell diameter above 30 µm (43).
