# Supplementary material for: The Transcription Factor Pdr802 Regulates Titan Cell Formation and Pathogenicity of Cryptococcus neoformans
Source: mBio. 2021 Mar 9;12(2):e03457-20. doi: 10.1128/mBio.03457-20 (PMC8092302; doi:10.1128/mBio.03457-20)
Supplement: FIG S7 [file mBio.03457-20-sf007.pdf]

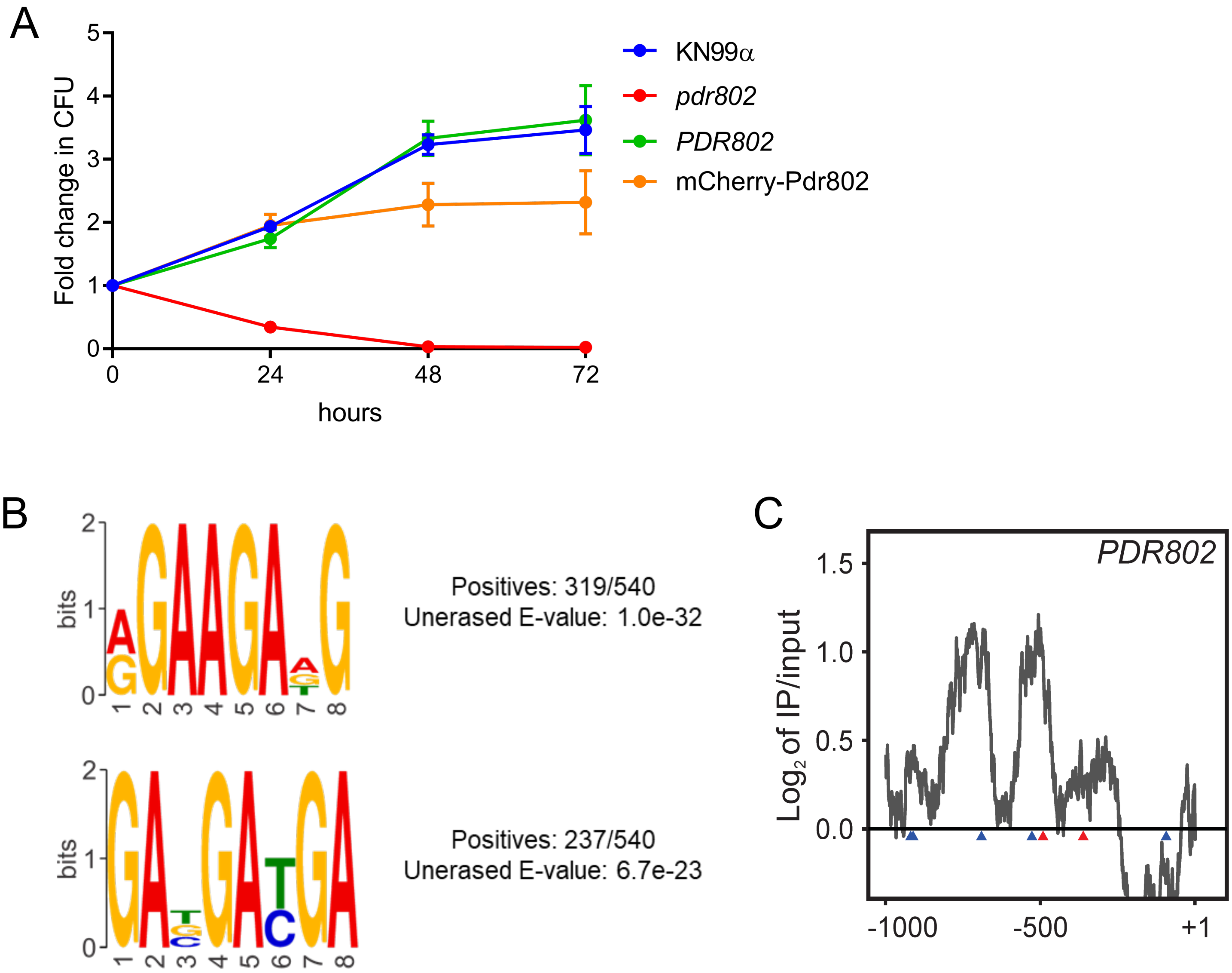

Figure S7. Strain viability, Pdr802 interaction with its own promoter, and putative DNA-binding motifs. A. The indicated strains were grown in DMEM at 37°C and 5% CO_2 for the times shown and samples were tested for their ability to form colonies on YPD medium. Plotted is the fold-change in CFU relative to the initial culture. B. Putative Pdr802-binding motifs determined using DREME (76). Primary and secondary hits are shown for analysis of 1,000 bp upstream of the initiating ATG. C. The ratios (log_2) of reads from immunoprecipitated (IP) DNA to reads from input DNA were calculated for 1,000 bp upstream of the first coding nucleotide (+1) of *PDR802*; shown is the difference in these values between tagged and untagged strains. Red triangles, complete Pdr802 DNA-binding motifs (Figure S7C); blue triangles, partial motifs.
