# Supplementary material for: The Transcription Factor Pdr802 Regulates Titan Cell Formation and Pathogenicity of Cryptococcus neoformans
Source: mBio. 2021 Mar 9;12(2):e03457-20. doi: 10.1128/mBio.03457-20 (PMC8092302; doi:10.1128/mBio.03457-20)
Supplement: TEXT S1 [file mBio.03457-20-s0001.pdf]

SUPPLEMENTARY METHODS

Strain construction

To complement the *pdr802* deletion mutant at its native locus, we used a split marker strategy (1) to replace the nourseothricin marker with the original *PDR802* sequence (including its native promoter and terminator) followed by a geneticin (G418) resistance marker. To do this we PCR amplified the *PDR802* sequence (4131 bp) with primers JR01/02 and the G418 resistance marker (1627 bp) with primers JR03/04 (see the Data Set S2, Sheet 5 for primer sequences); both amplicons were then gel purified and cloned in tandem into NdeI-digested pUC19, using Gibson Assembly Master Mix (2), to form pUC19+PDR802+G418. This plasmid was digested with NdeI and BssHII to release a 5492-bp fragment containing *PDR802* and a 5' portion of the G418 marker. The 3' portion of the G418 resistance marker (750 bp) was amplified using primers JR05/06 and fused by PCR to a region 3' of *PDR802* (1017 bp; amplified with JR07/08) to yield a 1767 bp product. These fragments were used in biolistic transformation of *pdr802* cells as in ref (3).

To tag Pdr802 with mCherry at the N-terminus, we first fused approximately 1.2 kb upstream of the start codon of *PDR802,* a sequence encoding mCherry, the *PDR802* coding sequence with 500 bp of downstream sequence, and a NAT resistance marker sequence. To generate these segments the *PDR802* promoter (1286 bp) was amplified with primers JR09/10; the mCherry sequence (714 bp) with JR11/12; the *PDR802* coding sequence (2957 bp) with JR13/14; and the NAT marker cassette (1721 bp) with primers JR15/16. All fragments were gel purified and cloned as above into pUC19 digested with Sac1 and Nde1, to form pUC19+mCherry+Pdr+NAT. This was digested with NdeI and BssHII to release a 5559 bp fragment. The 3' portion of the NAT resistance marker (832 bp) was amplified with primers JR17/18 and fused by PCR to a region 3' of *PDR802* amplified with JR19/20 (1064 bp) to form a 1896-bp product. These fragments were used in biolistic transformation of KN99α cells as above. All strains were confirmed by PCR and sequencing, and further analyzed by RT-PCR and qRT-PCR (see Supplemental Figure 1).

qRT-PCR

RNA from cryptococcal cells grown in DMEM at 37°C with 5% CO_2 for 24 hours was extracted with TriZol® reagent (Invitrogen, MA, USA) according to the manufacturer's instructions. RNA integrity was assessed by electrophoresis on 1% agarose and quantification was performed by absorbance analysis using a NanoDrop™ 2000 spectrophotometer (Thermo Fisher Scientific). cDNAs were prepared from DNase (Promega, WI, USA)-treated total RNA samples (290 ng) using ImProm-II reverse transcriptase (Promega) and oligo-dT. Quantitative real-time PCR (qRT-PCR) was performed on a Fast 7500 real-time PCR system (Applied Biosystems, MA, USA) with the following thermal cycling conditions: 95°C for 10 min followed by 40 cycles of 95°C for 15 s, 55°C for 15 s, and 60°C for 60 s. Platinum® SYBR® green qPCR Supermix (Invitrogen) was used as the reaction mix and supplemented with 5 pmol of each primer (see Data Set S2, Sheet 5, for sequences) and 8 ng of cDNA template for a final volume

of 10 µl. All experiments were performed in biological triplicate and each sample was analyzed in triplicate for each primer pair. Melting curve analysis was performed at the end of the reaction to confirm the presence of a single PCR product. Data were normalized to levels of *ACT1*, which was included in each set of PCR experiments. Relative expression was determined using the $2^{-\Delta Ct}$ method.

REFERENCES

1. Fu J, Hettler E, Wickes BL. 2006. Split marker transformation increases homologous integration frequency in *Cryptococcus neoformans*. Fungal Genet Biol 43:200–212.
2. Gibson DG, Young L, Chuang R-Y, Venter JC, Hutchison CA, Smith HO. 2009. Enzymatic assembly of DNA molecules up to several hundred kilobases. Nat Methods 6:343–345.
3. Friedman RZ, Gish SR, Brown H, Brier L, Howard N, Doering TL, Brent MR. 2018. Unintended Side Effects of Transformation Are Very Rare in *Cryptococcus neoformans*. G3 (Bethesda) 8:815–822.
